# Imatinib induces ferroptosis in gastrointestinal stromal tumors by promoting STUB1-mediated GPX4 ubiquitination

**DOI:** 10.1038/s41419-023-06300-2

**Published:** 2023-12-18

**Authors:** Xiangfei Sun, Qiang Zhang, Xiaohan Lin, Ping Shu, Xiaodong Gao, Kuntang Shen

**Affiliations:** 1 Department of General Surgery, Zhongshan Hospital, Fudan University School of Medicine, Shanghai, 200032 China; 2 Department of General Surgery, The First Affiliated Hospital of Nanjing Medical University, Nanjing, 210029 China

**Keywords:** Ubiquitylation, Prognostic markers

## Abstract

Imatinib (IM) has significantly improved the prognosis of gastrointestinal stromal tumor (GIST) patients, but some patients still have primary resistance to IM, and approximately half of patients develop acquired drug resistance within 2 years of treatment, necessitating exploration of new treatment strategies. Targeting ferroptosis as a novel approach to tumor treatment has gained attention. Yet, there is limited research on ferroptosis in GIST, and the underlying mechanism remains unclear. In this study, we revealed that IM increased lipid reactive oxygen species and intracellular Fe^2+^ levels, and decreased glutathione levels in GIST. This effect could be partially inhibited by Ferrostatin-1. Additionally, knocking down STUB1 and overexpressing GPX4 reversed the IM-induced ferroptosis effect. Moreover, STUB1 was identified as a novel E3 ubiquitin ligase of GPX4, promoting the ubiquitination at site K191 of GPX4. The combination of the GPX4 inhibitor RSL3 and IM synergistically induces ferroptosis, inhibiting GIST proliferation both in vivo and in vitro. Furthermore, STUB1 and GPX4 expression serve as independent prognostic factors for GIST. In conclusion, This study is the first to demonstrate that IM induces ferroptosis by promoting STUB1-mediated GPX4 ubiquitination in GIST, and the combination of RSL3 and IM emerges as a promising therapeutic strategy for GIST.

## Introduction

Gastrointestinal stromal tumor (GIST) is the most common tumor of mesenchymal origin in the gastrointestinal tract, with an annual incidence of 1/100,000–2/100,000 [1]. C-kit mutations are detected in 75%-80% of patients, whereas platelet-derived growth factor receptor alpha (PDGFRA) gene mutations only account for 5%-10% [2]. Early diagnosis of GIST is often difficult. Surgical resection remains the most effective treatment, but recurrence and metastasis often occur postoperatively. In addition, GIST is insensitive to radiotherapy and chemotherapy, making the prognosis poor. Although the emergence of tyrosine kinase inhibitors (TKIs) has significantly improved the prognosis of GIST patients, including Imatinib (IM) as the first-line treatment for recurrent, metastatic, and unresectable GIST [3], about 50% of GIST patients may develop acquired drug resistance within 2 years of treatment [4]. Although TKIs have been developed to the fourth generation, acquired drug resistance remains a problem. As a result, the overall treatment outcome of GIST remains unsatisfactory, and the latest clinical and basic research has focused on the new therapeutic targets [5].

Ferroptosis, a regulated cell death caused by fatal lipid peroxidation was proposed by Dixon in 2012 [6]. Subsequent studies identified lipid peroxidation as a key factor triggering membrane oxidative damage during ferroptosis [7]. Lipid peroxides are the result of oxidative damage to polyunsaturated fatty acids (PUFAs) caused by reactive oxygen species (ROS). This oxidative damage can disrupt the lipid bilayer and impact membrane function, ultimately leading to ferroptosis [8]. Inside the cell, the clearance of lipid peroxides primarily relies on the activity of the antioxidant enzyme glutathione peroxidase 4 (GPX4). GPX4 utilizes glutathione (GSH) as a substrate to convert lipid peroxides into normal phospholipid molecules. The synthesis of GSH is contingent on the availability of intracellular cysteine, which can be generated from cystine imported from the extracellular space via the cysteine-glutamate antiporter (Systemxc-, composed of recombinant solute carrier family 7, member 11, SLC7A11, and recombinant solute carrier family 3, member 2, SLC3A2) [9]. Because GPX4 is the sole cellular enzyme capable of reducing lipid peroxides to lipids, it plays a critical role in ferroptosis and serves as the target for various ferroptosis inducers [10]. A variety of tumors have been reported to be associated with ferroptosis, and targeting ferroptosis against tumor development and progression has become a promising approach in tumor therapy [11]. Several drugs have been identified to act through ferroptosis induction in different tumors, mainly including Systemxc- inhibitors (Erastin, sulfasalazine, sorafenib), glutathione depletion factor (FIN56), and GPX4 inhibitors (RSL3). Notably, sulfasalazine and sorafenib have received approval from the US Food and Drug Administration (FDA) for clinical use [12–16]. However, the existence of ferroptosis in GIST and its mechanism remain unclear.

The ubiquitin-proteasome pathway regulates target protein degradation, involving ubiquitin-activating enzyme 1 (E1), ubiquitin-conjugating enzyme 2 (E2) and ubiquitin ligase enzyme 3 (E3). Dysfunctional ubiquitin E3 ligase expression determines ubiquitination specificity and rate-limiting steps, playing a crucial role in tumor development [17, 18]. STUB1, also known as the C terminus of HSP 70 interacting protein (CHIP), contains a tetratricopeptide repeat and a U-box, which is a ubiquitin E3 ligase targeting various oncogene-encoded proteins (such as p53, c-Myc, PTEN, AR-V7, and EGFR) [19–22].

This study is the first to demonstrate that IM induces ferroptosis by promoting STUB1-mediated GPX4 ubiquitination in GIST and identify STUB1 as a novel ubiquitin E3 ligase targeting GPX4. Moreover, we demonstrate the synergistic effect of IM combined with RSL3 in treating GIST, suggesting that inhibiting GPX4 to induce ferroptosis could enhance GIST therapy.

## Materials and methods

### Cell culture

GIST-T1 cell line was purchased from Cosmo Bio Co. Ltd. (Tokyo, Japan) and GIST-882 cell line was provided by Dr. Fletcher of Harvard Medical School. Both cell lines were verified by short tandem repeat (STR) profiling and tested negative for mycoplasma contamination. Human GIST cell lines (GIST-T1 and GIST-882) were cultured in Iscove’s Modified Dulbecco Medium (IMDM, Corning, #10–016-CV, USA) and RPMI 1640 medium (Gibco, #11875119, USA) supplemented with 10% fetal bovine serum (FBS, Gibco, #10099141C, USA) and 1% penicillin-streptomycin (Gibco, #15140122, USA). Cells were passaged at 70%–80% confluence by dissociation from plates using 0.25% Trypsin-EDTA (Gibco, #25200056, USA). All cells were incubated in a 37°C incubator (Forma, USA) containing 5% CO_2_.

### Cell viability assay

The cells were seeded in a 96-well plate with 2 × 10^3^ cells and 100 µl medium per well. After culturing for some time, the medium was replaced with 100 µl medium with 10 µl cell counting kit-8 (#40203ES76, CCK-8, Yeasen, China). After 2 hours, the 96-well plate was oscillated for 2 min. The absorbance value was detected with a microplate reader (Thermo Fisher, USA) at 450 nm. Three replicate wells were run in each experiment, and each experiment was repeated three times. The cell viability results were normalized to the number of cells in the control group. Drug inhibition assay was performed by seeding and incubating 5 × 10^3^ GIST-T1 or GIST-882 cells at 37 °C overnight. Cells were treated with RSL3 and IM at various doses, and the half-maximal inhibitory concentration assay (IC50) was calculated using nonlinear regression analysis in GraphPad Prism 8.0.2. The synergistic effect of the combination treatment was measured using CompuSyn software (Paramus, NJ, USA). The combination index (CI) >1, =1, and <1 indicate antagonistic, additive, or synergic effects, respectively.

### Cell transfection

To knock down STUB1, we transfected cells with siRNA duplex oligos in OPTI-MEM (#2185849, Gibco) overnight. Expression plasmids encoding Myc-STUB1 (#90977–1), Flag-GPX4 (#68273–1), HA-ubiquitin (Ub, #61618–1), GPX4-K107R (#68273–2), GPX4-K126R (#68273–3), GPX4-K162R (#68273–4), GPX4-K167R (#68273–5), and GPX4-K191R (#68273–6) (GPX4 mutants with lys107, lys126, lys162, lys167 and lys191 changed into arginine) were purchased from Genechem (Genechem, Shanghai, China). All plasmids were transfected with Lipofectamine™ 3000 Transfection Reagent (#L3000015, ThermoFisher, Shanghai, China) according to the protocol.

### Viruses and transduction

STUB1 and GPX4 expression lentiviruses were purchased from Genechem (Genechem, Shanghai, China). Transfection was performed according to the manufacturer’s protocols. The transfected cells were screened by puromycin (2 mg/mL) (ST551, Beyotime, Beijing, China) to establish a stably expressing cell line (Fig. S1).

### Transmission electron microscopy

Cells were resuspended in a buffer containing 4% paraformaldehyde and 1% glutaraldehyde and subsequently dehydrated through graded ethanol and propylene oxide, which was then embedded in Epon 812 and sliced into 60-nm ultrathin sections. The mitochondrial area and structure were finally assessed by transmission electron microscopy (H-7650, Hitachinaka, Japan).

### Intra-cellular reactive oxygen species and lipid reactive oxygen species assay

ROS or lipid ROS levels were measured in the GIST-T1 and GIST-882 cells treated with IM (50 nM for T1 and 200 nM for 882) or Ferrostatin-1 (1 μM) for 12 h, by adding 10 µM 2ʹ,7ʹ-dichlorofluorescein diacetate (DCFH-DA) (ab113851, ROS Detection Assay Kit, Abcam) or 10 µM C11-BODIPY (#D3861, Thermo Fisher Scientific) for 30 min at 37 °C, followed by trypsinization, washing and resuspension in phosphate buffered saline (PBS). ROS and lipid ROS levels were then measured by confocal microscopy (Carl Zeiss, Jena, Germany) or flow cytometry. Flow cytometry analysis was performed using the SONY SH800 Cell Sorter with the FITC filter for oxidized C11-BODIPY (emission: 510 nm) and the PE-TexasRed filter for reduced C11-BODIPY (emission: 590 nm). At least 10,000 cells were collected for each condition, and this experiment was independently repeated three times. Data analysis was carried out using the FlowJo software (version 9.4.10, Tree Star). The results are expressed as the ratio of oxidized to reduced C11-BODIPY.

### Measurement of intracellular Fe^2+^ levels

Change of intracellular Fe^2+^ in GIST cells was detected with the novel fluorescent probe FerroOrange (F374, Dojindo) according to the manufacturer’s protocol. GIST-T1 and GIST-882 cells treated with IM were incubated with 1 µM FerroOrange at 37 °C for 30 min. The fluorescence was then measured by flow cytometry.

### GSH assay measurement

GSH and GSSG levels were determined using the GSH and GSSG Assay Kit (Beyotime, S0053) following the manufacturer’s instructions. To prepare the samples, GIST-T1 and GIST-882 cells were mixed with protein removal reagent M in a three-fold volume and thoroughly vortexed. Subsequently, two freeze-thaw cycles (5 s freeze, 1 min thaw) were performed alternating between liquid nitrogen and a 37 °C water bath. After centrifugation at 10,000 g for 10 min at 4 °C, the resulting supernatant was collected as the sample solution. All samples were normalized based on their protein content. Then, the 0.2 ml sample solution was mixed with 1 mM GSH solution of the same volume and incubated at 37 °C for 5 min; after addition of 0.1 ml reagent 1 and incubation at 37 °C for another 5 min, 2 ml reagent 2 was added. The mixture was then centrifuged at 10,000 g for 10 min. The supernatant (1 ml) was extracted and mixed with 1 ml reagent 3, 0.25 ml reagent 4 and 0.05 ml reagent 5, incubated for 15 min. Supernatants from each group of samples were collected, and the quantification of total GSH (GSH + GSSG) was performed by monitoring the formation of 2‐nitro‐5‐thiobenzoic acid at 412 nm. To determine GSSG specifically, the supernatants were derivatized using 1‐methyl‐2‐vinylpyridium trifluoromethane sulfonate. Standard curves were established for both GSH and GSSG, and the concentration of GSH was calculated by subtracting the GSSG concentration from the total GSH (GSH + GSSG). The measured concentrations of GSH and GSSG were then expressed as a ratio (GSH/GSSG).

### Quantitative real-time polymerase chain reaction

Total RNA was extracted using Trizol (Yeasen Biotechnology Shanghai, China) and reverse-transcribed into cDNA. quantitative real-time polymerase chain reaction was carried out using the SYBR Green kit. The relative mRNA expression was performed using the 2^-∆∆Ct^ method. GAPDH was used as internal control and the primers used are specified in Table S1.

### Protein stability assay

Protein stability was determined by using the cycloheximide (CHX) chase assay. Cells were treated with 100 μg/mL CHX for various time intervals after overexpression of STUB1. Then GPX4 protein lysates were resolved on SDS-PAGE and analyzed by Western blotting.

### Immunoprecipitation assays

Co-immunoprecipitation (CO-IP) assay was performed using the protein binding IP kit (abs955, Absin). Cell lysates were first incubated overnight with anti-STUB1 (Abcam), anti-GPX4 (Proteintech), or control IgG (Cell Signaling Technology) at 4 °C overnight, and then incubated with protein A/G-agarose beads at 4 °C for 4 h. The precipitates were washed five times in a wash buffer and evaluated using Western blot analysis.

### Western blot analysis

Western & IP lysis buffer (P0013, Beyotime) supplemented with the protease inhibitor and phosphatase inhibitors (#524625 and #539131, Millipore) was used to lyse cells on ice for 30 min. The PVDF membranes (ISEQ00010, Millipore) were blocked with 5% skimmed milk at room temperature for 1 h, followed by incubation with primary antibodies at 4 °C overnight. Then the membranes were washed 5 times for 5 min with TBST (TBS with 0.1% Tween 20) and incubated with HRP-conjugated secondary antibodies (CST, MA, USA) at room temperature for 1 h. The antibodies and the concentrations used are listed in Table S2. The membrane was then washed and visualized using enhanced chemiluminescence (ECL) solution (Millipore, Beverly, USA) and analyzed using ImageJ software (Version 1.53, National Institutes of Health, Bethesda, MD).

### Tissue microarray and immunohistochemistry

Tissue microarray (TMA) containing 418 paraffin-embedded primary GIST surgical samples resected at our hospital between January 2014 and January 2021 with Institutional Review Board approval were constructed. Informed consent was obtained from all subjects. Immunohistochemistry (IHC) staining was performed with anti-STUB1 and anti-GPX4 antibodies. The antibodies and the concentrations used are listed in Table S2. The staining intensity and the proportion of positively stained cells were evaluated using Image J 1.53 software. The staining intensity was graded as follows: negative = 0, mild = 1, moderate = 2, and strong = 3. The proportion of positively stained cells was scored as follows: 1 = 0–24% positive cells; 2 = 25–49% positive cells; 3 = 50–74% positive cells; and 4 = 75%-100% positive cells. The IHC staining score was expressed by the intensity multiplied by the proportional score. A staining score of <8 was regarded as the low expression group and that ≥8 as the high expression group.

### Immunofluorescence staining

Cultured cells were fixed using 4% paraformaldehyde (Sigma-Aldrich, #30525–89–4) in PBS for 20 min. Subsequently, they were permeabilized with 0.3% Triton X-100 (Sigma-Aldrich, #9036–19–5) in PBS for 10 min and then blocked with 10% normal goat serum (Beyotime, #C0265, Jiangsu, China) for 1 h at 37 °C. This was followed by overnight incubation at 4 °C with primary antibodies against STUB1 (Abcam, #ab134064, 1:250) and GPX4 (Abcam, #ab125066, 1:200). Afterwards, the cells were washed three times with PBS containing Tween 20 and then incubated with fluorescent secondary antibodies for 1 h at 37 °C. The secondary antibody for STUB1 was green fluorescent, and the secondary antibody for GPX4 was red fluorescent. Subsequent to this, the cells were rinsed three times with PBS and cell nuclei were stained with 1 μg/ml DAPI (Sigma-Aldrich, #D9542) for 10 min. Image acquisition was performed using Zeiss LSM780 Laser Scanning Confocal Microscopy (Carl Zeiss, Jena, Germany). To quantitatively analyze colocalization, ImageJ software (Version 1.53, National Institutes of Health, Bethesda, MD) was utilized in conjunction with the Colocalization Finder plugin for calculating Pearson’s correlation coefficients [23].

### In situ proximity ligation assay

GIST cells were initially seeded in 6-well CultureSlides obtained from BD Biosciences (Mountain View, CA, USA). The cells were then fixed using 4% paraformaldehyde for a duration of 15 min and subsequently permeabilized with a solution consisting of 0.5% Triton X-100 in PBS for 30 min. The slides were further processed for an in situ Proximity Ligation Assay following the manufacturer’s instructions, employing the Duolink® II Detection Reagents Green, Duolink® II PLA probe anti-Rabbit Minus, and Duolink® II PLA probe anti-Mouse Plus (Olink Bioscience, Uppsala, Sweden). Primary antibodies used were STUB1 (Abcam, #ab134064) and GPX4 (Proteintech, #67763–1-Ig). The resulting images were captured using Zeiss LSM780 Laser Scanning Confocal Microscopy (Carl Zeiss, Jena, Germany). Subsequent quantification was performed using ImageJ software (Version 1.53, National Institutes of Health, Bethesda, MD).

### Mouse xenograft study

Animal studies were reviewed and approved by the Institutional Review Boards of Zhongshan Hospital. GIST-T1 xenografts were established in 6-week-old male BALB/c nude mice (Shanghai Jiesijie Laboratory Animal Corporation) by inoculating 5 × 10^6^ cells mixed with Matrigel (BD Biosciences) at a 1:1 ratio (volume) into the dorsolateral side. When the tumor reached 50mm^3^, the mice were assigned randomly into DMSO, IM, RSL3, and IM combined with RSL3 groups. IM was dissolved in dimethyl sulfoxide (DMSO, #HY-Y0320, MCE), and diluted in PBS. RSL3 was dissolved in DMSO, diluted in corn oil (#HY-Y1888, MCE), and then intraperitoneally injected into mice with IM (100 mg/kg) or RSL3 (10 mg/kg) every 3 days. The dosages of IM and RSL3 were determined based on previous studies [24, 25] and our own preliminary experimental results. Tumor sizes were measured every 3 days, and tumor volumes were calculated as follows: 0.5×length×width^2^. Each group consisted of 6 mice. All mice were euthanized after 18 days of treatment, and the tumors were surgically excised. A portion of the tumors was immediately fixed in 4% paraformaldehyde, and sectioned for histological hematoxylin–eosin (HE) (Zhongshanjinqiao, Beijing, China) and IHC staining. Experimenters were not blind to experimental groups. No pre-test analyses were used to estimate sample sizes. No data were excluded from the final analyses.

### Reagents

The reagents used and their commercial sources are indicated as follows: Dimethyl sulfoxide (#HY-Y0320), Corn oil (#HY-Y1888), Imatinib Mesylate (IM) (#HY-50946), RSL3 (#HY-100218A), Ferrostatin-1 (#HY-100579), MG-132 (#HY-13259) and Cycloheximide (CHX, #HY-12320), all of which were purchased from Med-Chem-Express (MCE; Shanghai, China).

### Statistical analysis

Categorical variables are expressed as percentages and compared using the *χ*2 test and Fisher’s exact tests when appropriate, while continuous variables were presented as means ± standard deviation (SD) and compared using a double-tailed Students’ *t* test. The number of animals and experimental repeats is listed in individual legends. Data meets the assumptions of the tests. The sample variation within each group was estimated to ensure that the variance is similar. All statistical tests were two-sided and statistically significance was declared when the *P*-value was ≤ 0.05. Error bar = mean ± standard deviation in all graphs. Statistical analyses were performed using GraphPad Prism (version 8.0.2, San Diego, USA).

## Results

### The ferroptosis inhibitor Ferrostatin-1 rescues GIST cells treated with IM

**Fig. 1:**
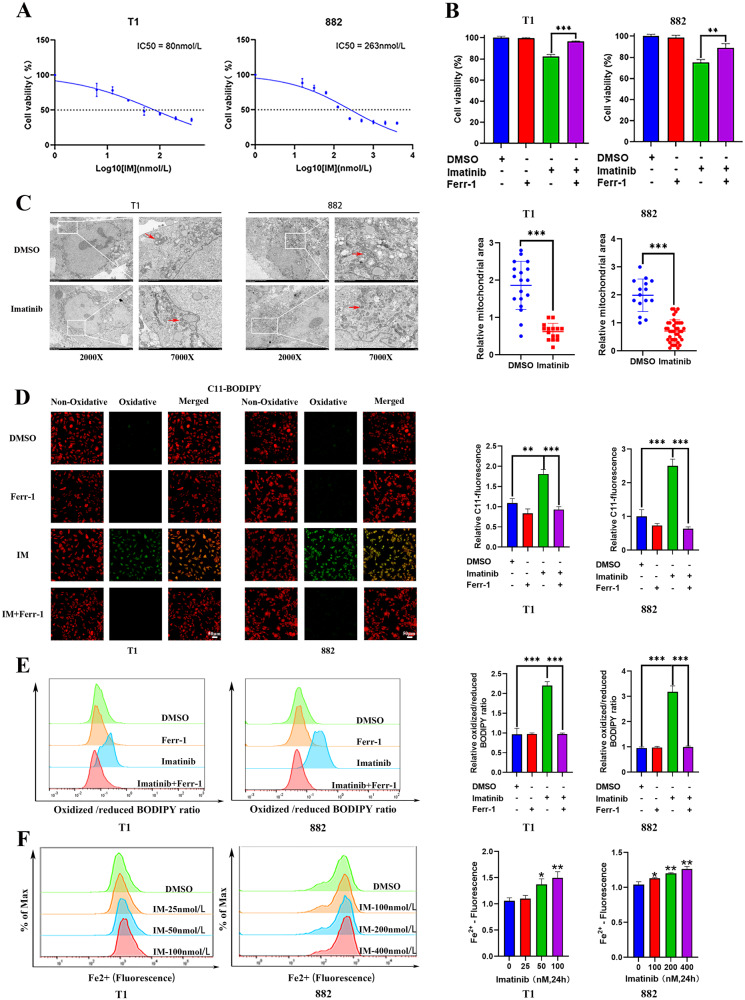
Imatinib (IM) induces ferroptosis in gastrointestinal stromal tumor (GIST) cells. **A** GIST-T1 and 882 cells were treated with different concentrations of IM for 24 h and the IC50 of IM was determined. **B** GIST-T1 and 882 cells were treated with IM (50 nM for T1 and 200 nM for 882) for 24 h in the absence or presence of Ferrostatin-1 (1 μM), and the cell viability was assayed. **C** Cell morphology was observed via transmission electron microscopy after the cells were treated with IM (50 nM for T1 and 200 nM for 882) for 24 h. The area of mitochondria are quantitatively analyzed by using the ImageJ software. **D**, **E** GIST-T1 and 882 cells were treated with IM (50 nM for T1 and 200 nM for 882) in the absence or presence of Ferr-1 (1 μM) for 12 h, the relative lipid ROS levels were measured using a C11-BODIPY lipid peroxidation sensor via confocal microscope (**D**) and flow cytometry (**E**). In fluorescence images, the increase in lipid ROS levels resulted in the oxidation of the polyunsaturated butadienyl portion of C11-BODIPY, causing a shift in fluorescence from red to green. Flow cytometry analysis utilized the FITC filter for oxidized C11-BODIPY (emission: 510 nm) and the PE-TexasRed filter for reduced C11-BODIPY (emission: 590 nm). The results are presented as a ratio of oxidized to reduced C11-BODIPY. **F** GIST-T1 and 882 cells were treated with IM at 0, 25, 50, and 100 nM and 0, 100, 200, and 400 nM for 24 h, and the relative levels of Fe^2+^ were assayed. Scale bar, 50 μm in **D**. Experiments were independently repeated three times. Significance denoted by: ns not significant, *P < 0.05, **P < 0.01, and ***P < 0.001.

First, we investigated the sensitivity of GIST-T1 and GIST-882 cells to IM. The CCK-8 results showed that the IC50 was 80 nmol (GIST-T1) and 263 nmol (GIST-882) (Fig. 1A). The experimental doses of Ferrostatin-1 blocked the death of GIST-T1 and GIST-882 cells treated with RSL3, indicating the existence of ferroptosis in GIST (Fig. S2). Moreover, we examined whether Ferrostatin-1 could reverse the inhibitory effect of IM, and found that Ferrostatin-1 could partially rescue the inhibitory effect of IM on both GIST-T1 and GIST-882 cells (Fig. 1B).

### Ferrostatin-1 inhibits IM-induced ferroptosis in GIST cells

The detection indicators of ferroptosis include changes in mitochondrial morphology, lipid ROS levels, Fe^2+^ levels, and GSH levels. To clarify the relationship between IM and ferroptosis, we first investigated changes in the morphological characteristics of GIST cells after IM treatment by transmission electron microscopy. The results showed that the mitochondrial volume of GIST cells was reduced after IM treatment (Fig. 1C). Next, we measured the ROS and lipid ROS levels with the DCFH-DA or C11-BODIPY lipid peroxidation sensor. It was found that ROS and lipid-ROS levels in GIST-T1 and GIST-882 cells were increased after IM treatment and this effect could be reversed by Ferrostatin-1. However, Ferrostatin-1 itself did not affect ROS and lipid-ROS levels (Fig. S3 and 1D, E). In addition, The fluorescence intensity of FerroOrange was increased in GIST-T1 and GIST-882 cells treated with IM compared with that in untreated cells, illustrating that IM promoted the accumulation of Fe^2+^ in GIST (Fig. 1F). In conclusion, IM could induce ferroptosis in GIST cells.

### IM promotes ferroptosis by causing GPX4 protein degradation in GIST cells

**Fig. 2:**
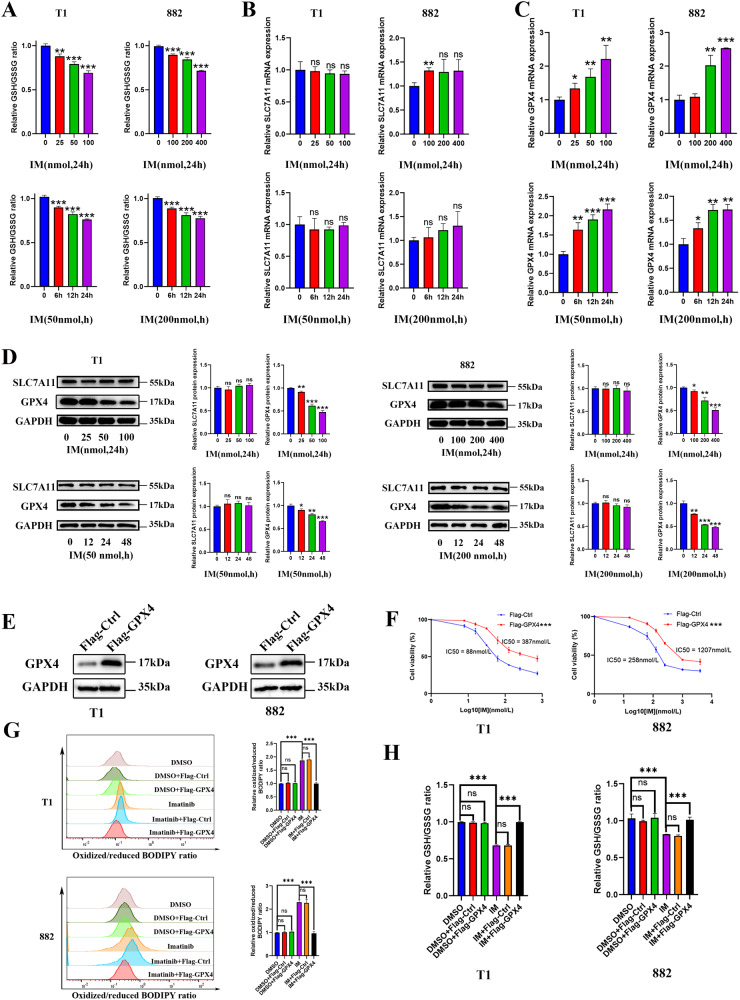
Imatinib (IM) induces ferroptosis by causing GPX4 degradation. **A** GIST-T1 and 882 cells were treated with IM at 0, 25, 50, and 100 nM and 0, 100, 200, and 400 nM for 24 h or treated with IM (50 nM for T1 and 200 nM for 882) for 0, 6, 12, or 24 h, and the relative GSH/GSSG ratios were assayed. **B** and **C** The relative RNA level of SLC7A11 and GPX4 was measured via quantitative real-time polymerase chain reaction after GIST-T1 and 882 cells were treated with IM at 0, 25, 50, and 100 nM and 0, 100, 200, and 400 nM for 24 h or treated with IM (50 nM for T1 and 200 nM for 882) for 0, 6, 12, or 24 h. **D** Representative Western blot analysis of SLC7A11 and GPX4 protein levels in GIST-T1 and 882 cells. The cells were treated with IM at 0, 25, 50, and 100 nM and 0, 100, 200, and 400 nM for 24 h or treated with IM (50 nM for T1 and 200 nM for 882) for 0, 12, 24, or 48 h. The histograms indicate the levels of the protein determined from 3 independent experiments expressed as the mean ratio relative to that in the untreated control after normalization to GAPDH. **E** Western blot analysis of GPX4 in GIST-T1 and 882 cells with GPX4 overexpression. **F** GIST-T1 and 882 cells were treated with IM (50 nM for T1 and 200 nM for 882) for 24 h with or without the overexpression of GPX4, and cell viability was assayed. **G** GIST-T1 and 882 cells were treated with IM (50 nM for T1 and 200 nM for 882) for 12 h with or without the overexpression of GPX4, and the relative lipid ROS levels were assayed via flow cytometry. Flow cytometry analysis was performed using the FITC filter for oxidized C11-BODIPY (emission: 510 nm) and the PE-TexasRed filter for reduced C11-BODIPY (emission: 590 nm). The results are displayed as a ratio of oxidized/reduced C11-BODIPY. **H** GIST-T1 and 882 cells were treated with IM (50 nM for T1 and 200 nM for 882) for 24 h with or without the overexpression of GPX4. The relative GSH/GSSG ratios were assayed. Experiments were independently repeated three times. Significance denoted by: ns not significant, *P < 0.05, **P < 0.01, and ***P < 0.001.

Knowing that ferroptosis caused by lipid peroxidation is regulated by the key components of the cysteine-glutamate antiporter known as systemxc- and the antioxidant enzyme glutathione peroxidase 4 (GPX4), we investigated how IM induced ferroptosis and the result showed that the GSH/GSSG ratio was decreased in GIST-T1 and GIST-882 cells treated with IM as compared with those in the untreated cells (Fig. 2A). To clarify the specific molecular mechanism of IM-induced ferroptosis, we examined the effects of different IM concentrations and treatment time on the expression of SLC7A11 and GPX4. The results showed no significant effect on the expression of SLC7A11 at the transcriptional level and protein levels (Fig. 2B, D). The expression of GPX4 increased in a dose- and time-dependent manner after IM treatment at the transcriptional level (Fig. 2C), whereas at the protein level, IM effectively induced the reduction of GPX4 protein level in GIST-T1 and GIST-882 cells (Fig. 2D). Therefore, IM may regulate GPX4 expression in a post-transcriptional manner. To further clarify the role of GPX4 in the anti-tumor effect of IM, we overexpressed GPX4 in GIST-T1 and GIST-882 cells to detect the change of IM sensitivity and its effect on lipid ROS and GSH/GSSG ratio (Fig. 2E). The results showed that GPX4 overexpression significantly reversed the anti-tumor effect of IM and the up-regulation of lipid ROS (Fig. 2F, G). In addition, GPX4 overexpression blocked the reduction of the GSH/GSSG ratio induced by IM (Fig. 2H). In conclusion, IM promotes ferroptosis in GIST by causing GPX4 protein degradation.

### IM promotes ferroptosis by regulating STUB1 expression in GIST cells

**Fig. 3:**
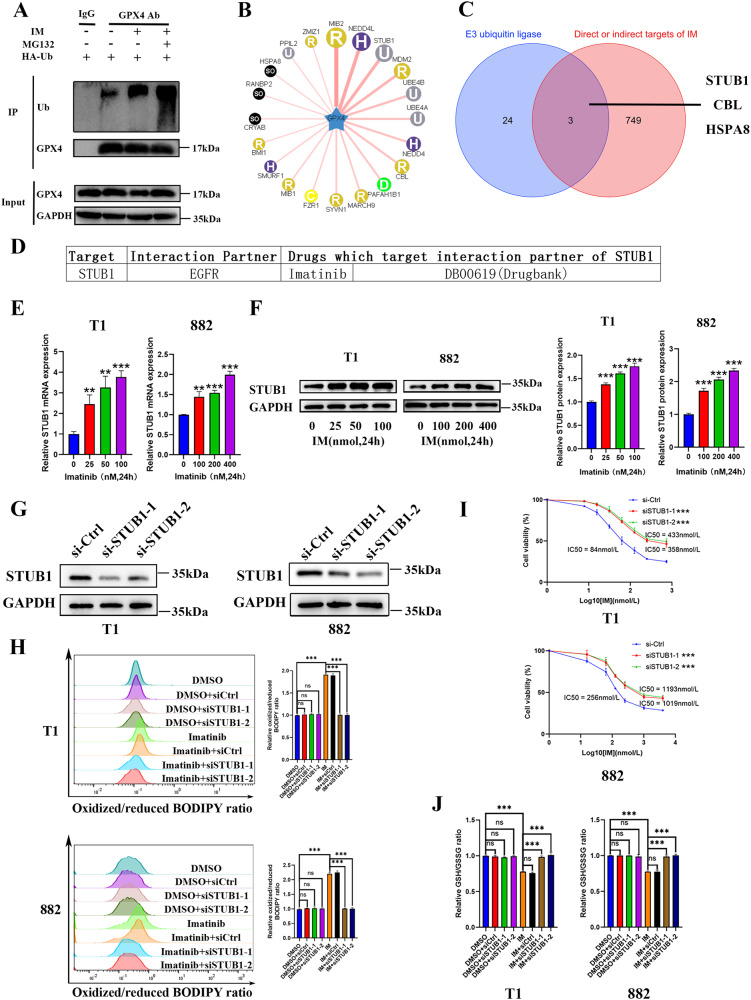
Imatinib (IM) induces ferroptosis by regulating the E3 ubiquitin ligase STUB1. **A** GIST-T1 cells were treated with IM (50 nM) for 24 h with or without MG132 (5 μM), and GPX4 ubiquitination was measured via CO-IP. **B** Bioinformatics prediction of the interaction between the GPX4 and multiple E3 ubiquitin ligases using the UbiBrowser website (http://ubibrowser.ncpsb.org.cn). **C** Venn diagram depicting that the predicted E3 ubiquitin ligase STUB1, CBL, and HSPA8 of GPX4 protein may be the targets of IM. **D** DRUGSURV database (http://www.bioprofiling.de/GEO/DRUGSURV/) analysis revealed that STUB1 is an indirect target of IM. **E** GIST-T1 and 882 cells were treated with IM at 0, 25, 50, and 100 nM and 0, 100, 200, and 400 nM for 24 h, and RNA expression of STUB1 were measured. **F** Representative Western blot analysis of STUB1 protein levels in GIST-T1 and 882 cells. The cells were treated with IM at 0, 25, 50, and 100 nM and 0, 100, 200, and 400 nM for 24 h, and subjected to Western blot analysis. The histograms indicate the levels of the protein determined from three independent experiments expressed as the mean ratio relative to that in the untreated control after normalization to GAPDH. **G** Western blot analysis of STUB1 in GIST-T1 and 882 cells transfected with siRNA-control, siRNA-STUB1–1 and siRNA-STUB1–2. **H** GIST-T1 and 882 cells were treated with IM (50 nM for T1 and 200 nM for 882) for 12 h with or without STUB1 knockdown, and the relative lipid ROS levels were assayed via flow cytometry. Flow cytometry analysis was performed using the FITC filter for oxidized C11-BODIPY (emission: 510 nm) and the PE-TexasRed filter for reduced C11-BODIPY (emission: 590 nm). The results are displayed as a ratio of oxidized/reduced C11-BODIPY. **I** GIST-T1 and 882 cells were treated with IM (50 nM for T1 and 200 nM for 882) for 24 h with or without STUB1 knockdown. Cell viability was assayed. **J** GIST-T1 and 882 cells were treated with IM (50 nM for T1 and 200 nM for 882) for 24 h with or without STUB1 knockdown. The relative GSH/GSSG ratios were assayed. Experiments were independently repeated three times. Significance denoted by: ns not significant, *P < 0.05, **P < 0.01, and ***P < 0.001.

To clarify the specific molecular mechanism of IM in promoting GPX4 protein degradation, we conducted CO-IP experiments to detect the effect of IM on the ubiquitination of GPX4 and the result showed that IM effectively induced the ubiquitination of GPX4 (Fig. 3A). Next, we used the ubiquitin ligase prediction website (http://ubibrowser.ncpsb.org.cn) to predict the ubiquitin ligase E3 of GPX4 (Fig. 3B and Table S3). Based on DRUGSURV database analysis (the first computational tool to estimate the potential effects of a drug) [26], we found that IM could indirectly target STUB1 (Fig. 3C, D). To detect the regulatory effect of IM on STUB1, we tested the effect of IM on STUB1 expression. The results showed that IM promoted STUB1 at both transcriptional and protein levels (Fig. 3E, F). Therefore, we speculated that STUB1 may play a role in IM-induced ferroptosis. To further clarify the effect of STUB1 on the anti-tumor activity of IM, STUB1 was knocked down in GIST-T1 and GIST-882 cells (Fig. 3G). The results showed that STUB1 knockdown effectively blocked the anti-tumor effect of IM and reduced the promotion of lipid-ROS mediated by IM (Fig. 3I, H). In addition, STUB1 knockdown blocked the reduction of the GSH/GSSG ratio induced by IM (Fig. 3J). Our results suggest that IM may promote ferroptosis by regulating STUB1 expression in GIST.

### STUB1 interacts with GPX4 and promotes GPX4 ubiquitination in GIST cells

**Fig. 4:**
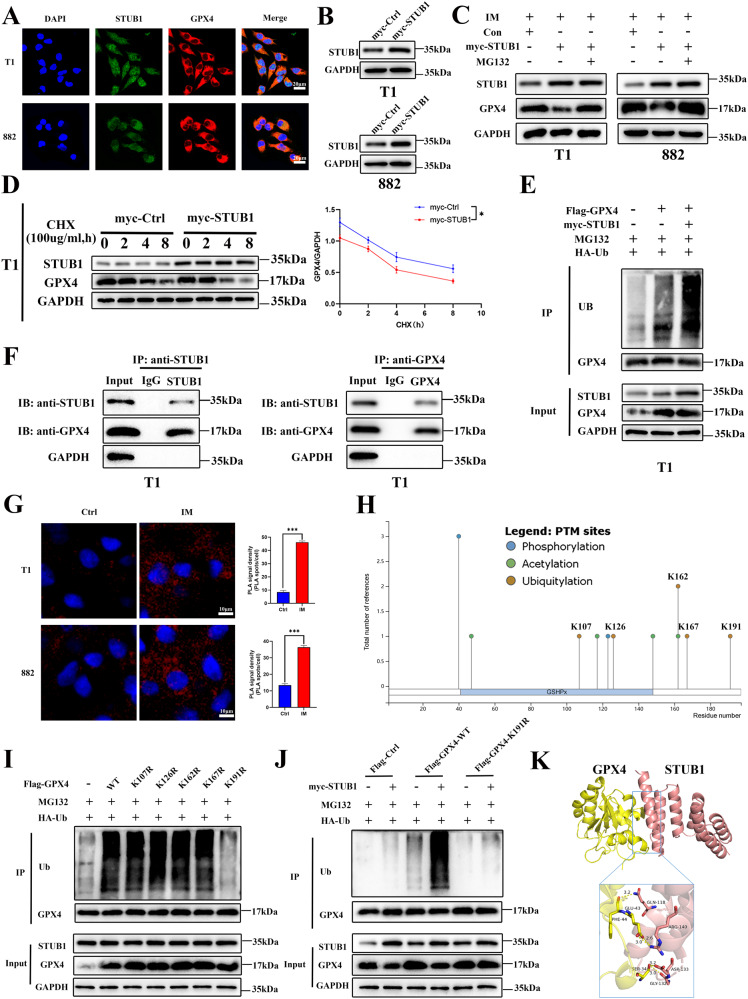
STUB1 interacts with GPX4 to promote GPX4 ubiquitination in gastrointestinal stromal tumor (GIST). **A** Localization of STUB1 (green) and GPX4 (red) in GIST-T1 and 882 cells by confocal laser scanning immunofluorescence. The nucleus is labeled by DAPI (blue). Colocalization of STUB1 (green) and GPX4 (red) in cells appears yellow in the merged images. **B** Western blot analysis of STUB1 in GIST-T1 and 882 cells transfected with STUB1 overexpression plasmids or control plasmid. **C** Western blotting was performed to assess the level of GPX4 in GIST-T1 cells transfected with GPX4 overexpression plasmids or control plasmid, and treated with IM (50 nM for T1 and 200 nM for 882) in the absence or presence of MG132 (5 μM) for 24 h. **D** STUB1 overexpression inhibited the stability of the GPX4 protein in GIST-T1 cells. The GPX4 protein levels were analyzed by western blotting after the cells were treated with CHX (100 μg/ml) for 0, 2, 4 or 8 h. **E** STUB1 overexpression promoted the ubiquitination of GPX4 protein in GIST-T1 cells. The ubiquitination of GPX4 protein was measured by CO-IP using an anti-ubiquitin antibody. **F** Direct interaction between STUB1 and GPX4 in GIST-T1 and 882 cells. Protein-protein interactions in GIST-T1 and 882 cells were validated by a CO-IP assay using an anti-STUB1 and an anti-GPX4 antibody. An antibody targeting IgG served as the negative control. **G** The representative images of proximity ligation assay (PLA). GIST-T1 and 882 cells were treated with IM (50 nM for T1 and 200 nM for 882) or a control vehicle for 24 h and then incubated with the anti-STUB1 and anti-GPX4 antibodies and detected by DuoLink probe (Olink Bioscience, Uppsala, Sweden). MG132 (5 μM) was added for stabilizing GPX4. The red dots indicate the positive PLA signal. **H** The prediction of the lysine site of ubiquitination of GPX4. **I** The effects of the wild-type and different mutation sites of GPX4 on ubiquitin modification. **J** The effect of STUB1 on the ubiquitination modification of the K191 site of GPX4. **K** Computational molecular docking simulation analyses of STUB1 and GPX4. Scale bar, 20 μm in **A**, 10 μm in **G**. Experiments were independently repeated three times. Significance denoted by: ns not significant, *P < 0.05, **P < 0.01, and ***P < 0.001.

Knowing that STUB1 and GPX4 play important roles in IM-induced ferroptosis, we first examined the localization of STUB1 and GPX4 in GIST cells to determine whether STUB1 was the E3 ubiquitin ligase of GPX4. The immunofluorescence result showed that STUB1 was present in the nucleus and cytoplasm, while GPX4 was predominantly located in the cytoplasm. Furthermore, there was co-localization of STUB1 and GPX4 in the cytoplasm of both GIST-T1 (Pearson correlation coefficient = 0.728 ± 0.024) and GIST-882 cells (Pearson correlation coefficient = 0.689 ± 0.013; Figs. 4A and S4). First, we verified overexpression of STUB1 (Fig. 4B). Next, we further explored whether STUB1 affected the protein level of GPX4, and found that STUB1 overexpression further reduced the protein level of GPX4 under IM treatment. However, STUB1 overexpression was no longer able to reduce the expression of GPX4 after treatment of the cells with the proteasome inhibitor MG132 (Fig. 4C). Then we further explored whether STUB1 regulates GPX4 stability via the ubiquitination of GPX4. It was found that STUB1 overexpression led to an increase GPX4 degradation and prolonged the half-life of GPX4 protein in GIST-T1 cells (Fig. 4D). Furthermore, STUB1 overexpression significantly increased GPX4 ubiquitination (Fig. 4E). To investigate whether STUB1 could directly regulate GPX4, protein-protein interaction was validated through a CO-IP assay using an anti-STUB1 and an anti-GPX4 antibody. The results showed that STUB1 could co-precipitate with GPX4 (Figs. 4F and S5). In addition, proximity ligation assay (PLA) revealed a close proximity between STUB1 and GPX4 proteins and IM could significantly promote the proximity between the two proteins in GIST-T1 and 882 cells (*P* < 0.001; Fig. 4G). To further explore the mechanism by which STUB1 promoted the ubiquitination and degradation of GPX4, PhosphositePlus database (https://www.phosphosite.org/homeAction) was used to speculate that the K107, K126, K162, K167, and K191 sites were potential ubiquitination modified amino acid sites of GPX4, and RCSB Protein Data Bank (https://www.rcsb.org/) was used to simulate the corresponding point mutation positions (Figs. 4H and S6). We then constructed the point mutation plasmids by mutating the lysine (Lys) to the arginine (Arg), which cannot be modified by ubiquitination, and performed CO-IP experiments. The results showed that among all the ubiquitination site mutants, only the K191R mutant had almost no detectable ubiquitination modification. Compared with other mutants, the ubiquitination level of the GPX4-K191R mutant was downregulated significantly when it was co-transfected with STUB1 (Fig. 4I, J). Computational molecular docking simulation analysis of STUB1 and GPX4 revealed a high possibility of combining structures (Fig. 4K). These findings collectively suggest that STUB1 is an E3 ligase of GPX4 that can promote its degradation by enhancing its ubiquitination.

### RSL3 exhibits synergistic inhibition with IM in vitro

**Fig. 5:**
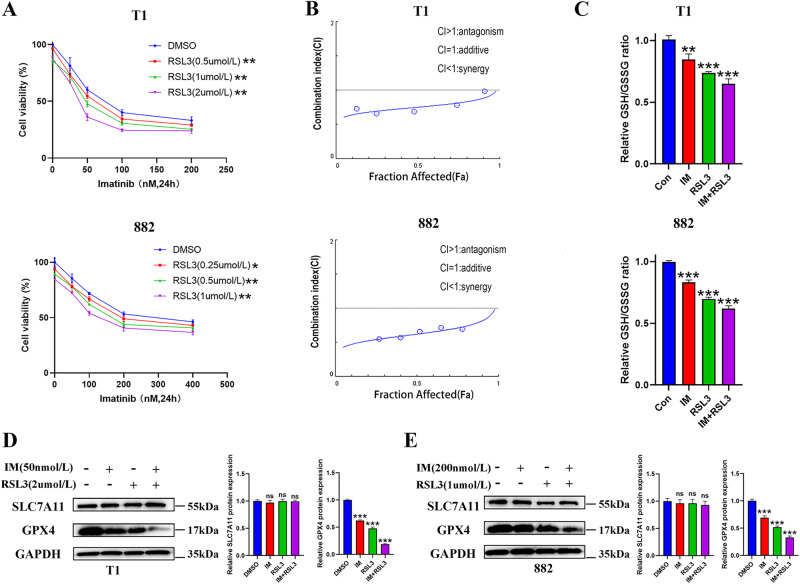
Combination treatment of Imatinib (IM) and RSL3 for gastrointestinal stromal tumor (GIST) in vitro. **A** CCK-8 viability assay following treatment for 24 h with escalating concentrations of IM (0, 25, 50, 100, and 200 nM for T1 and 0, 50, 100, 200, and 400 nM for 882) and RSL3 (0, 0.5, 1, and 2 μM for T1 and 0, 0.25, 0.5 and 1 μM for 882) in GIST-T1 and 882 cells. **B** Synergy was calculated using CompuSyn and a combination index value of under 1.0 was considered synergy. **C** The relative GSH/GSSG ratios were assayed after GIST-T1 and 882 cells were treated with IM (50 nM for T1 and 200 nM for 882) and RSL3 (2 μM for T1 and 1 μM for 882) for 24 h. **D**, **E** Western blotting analysis of SLC7A11 and GPX4 protein expression after combination treatment of IM and RSL3 in GIST-T1 (**D**) and 882 cells (**E**) for 24 h. Experiments were independently repeated three times. Significance denoted by: ns not significant, *P < 0.05, **P < 0.01, and ***P < 0.001.

We evaluated the potential tumor suppressive effect of RSL3 in combination with IM in GIST-T1 and GIST-882 cells by treating with different concentrations of drug combinations. Our data revealed that a single treatment with IM or RSL3 significantly inhibited the GIST cell activity (Fig. 5A). The synergistic antitumor effect of RSL3 and IM was analyzed using CompuSyn software, which applies the median effect theorem of the law of mass action and its combination index theorem to facilitate pharmacodynamic studies and computerized analysis simulations. Combination index (CI) values were calculated based on the drug combination principles, as proposed by Chou—Talalay (CI <1, =1, and >1 indicate synergic, additive or antagonistic effects, respectively) [27]. Our results demonstrated that the CI values in both GIST-T1 and GIST-882 cells were <1, implying that RSL3 exerted synergistic effects with IM in both GIST-T1 and GIST-882 cells, offering an advantage of the combination treatment (Fig. 5B). The GSH/GSSG ratio (Fig. 5C) and the expression of GPX4 (Fig. 5D, E) in RSL3 combined with the IM group were lower than those in IM or RSL3 group alone. Thus, the combination of RSL3 and IM could synergistically inhibit GIST cell viability.

### RSL3 exhibits synergistic inhibition with IM in vivo

**Fig. 6:**
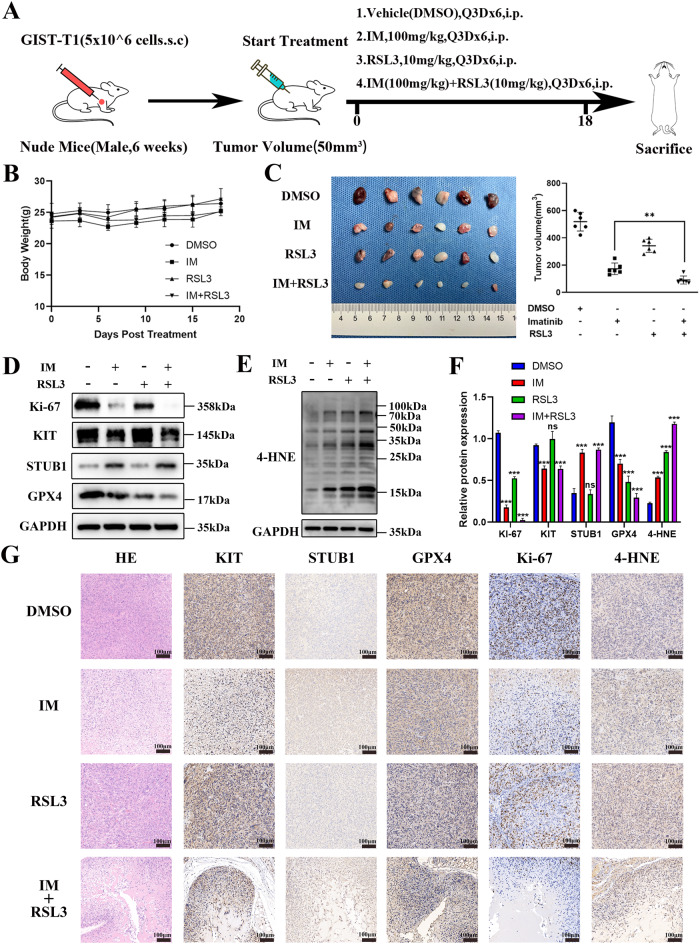
Subcutaneous tumor models of gastrointestinal stromal tumor (GIST) were established to analyze the synergy effect of Imatinib (IM) and RSL3. **A** GIST-T1 cells were subcutaneously transplanted into the dorsolateral side of nude mice. When the subcutaneous tumor volume reached 50 mm^3^, the mice were randomly divided into four groups (six mice in each group), including the vehicle (DMSO), IM (100 mg/kg), RSL3 (10 mg/kg) or IM combined with RSL3 treatment groups, and then were injected intraperitoneally every there day. All mice were euthanized 18 days later. **B** Body weight was measured every 3 days until day 18. **C** The images and quantitative analysis of tumor volume on day 18 after treatment. **D**–**F** Western blot analysis of the protein levels of Ki-67, KIT, STUB1, GPX4, and 4-HNE in GIST cell xenograft tumor tissues of mice from vehicle (DMSO), IM, RSL3, and combination treatment groups. *n* = 3 mice per group. **G** Immunohistochemical staining representative pictures of hematoxylin-eosin (HE), KIT, STUB1, GPX4, Ki-67, and 4-HNE in GIST cell xenograft tumor tissues of mice from vehicle (DMSO), IM, RSL3, and combination treatment groups. Scale bar, 100 μm. DMSO dimethyl sulfoxide, IM Imatinib, RSL3 Ras-selective lethal small molecule 3, 4-HNE 4-hydroxy-2-nonenal. Significance denoted by: ns not significant, *P < 0.05, **P < 0.01, and ***P < 0.001.

The GIST-T1 cells were subcutaneously implanted into the backs of immunodeficient nude mice. When the tumor volume reached 50 mm^3^, the mice were randomly divided into four groups and subsequently treated with either DMSO, IM, RSL3 or a combination of IM and RSL3 (Fig. 6A). No significant changes in body weight or signs of toxicity were observed in any of the treatment groups (Fig. 6B). Compared to the DMSO group, tumor growth was inhibited in both the IM and RSL3 groups, with the most significant inhibition observed in the IM combined with RSL3 group (Fig. 6C and S7). In addition, Western blotting analysis of KIT, STUB1, GPX4, Ki-67, and 4-HNE protein levels was performed on subcutaneous xenograft tumors. The results showed that the expression of KIT, Ki-67, and GPX4 were decreased and the expression of STUB1 and 4-HNE was increased in the IM-treated GIST-T1 xenografts, indicating that IM could induce ferroptosis in vivo. After treatment with RSL3, the expression of GPX4 and Ki-67 was decreased and the expression of 4-HNE was increased. In addition, the expression of Ki-67 and GPX4 was the lowest and the expression of 4-HNE was the highest in the combined-treated GIST-T1 xenograft model (Figs. 6D–F and S8). The representative IHC staining pictures of hematoxylin-eosin (HE), KIT, STUB1, GPX4, Ki-67, and 4-HNE are presented in Fig. 6G. These findings suggest that RSL3 enhances IM-induced lipid ROS and ferroptosis in GIST, leading to synergistic inhibition of GIST in vivo.

### STUB1 and GPX4 expressions are independent prognostic factors for GIST

**Fig. 7:**
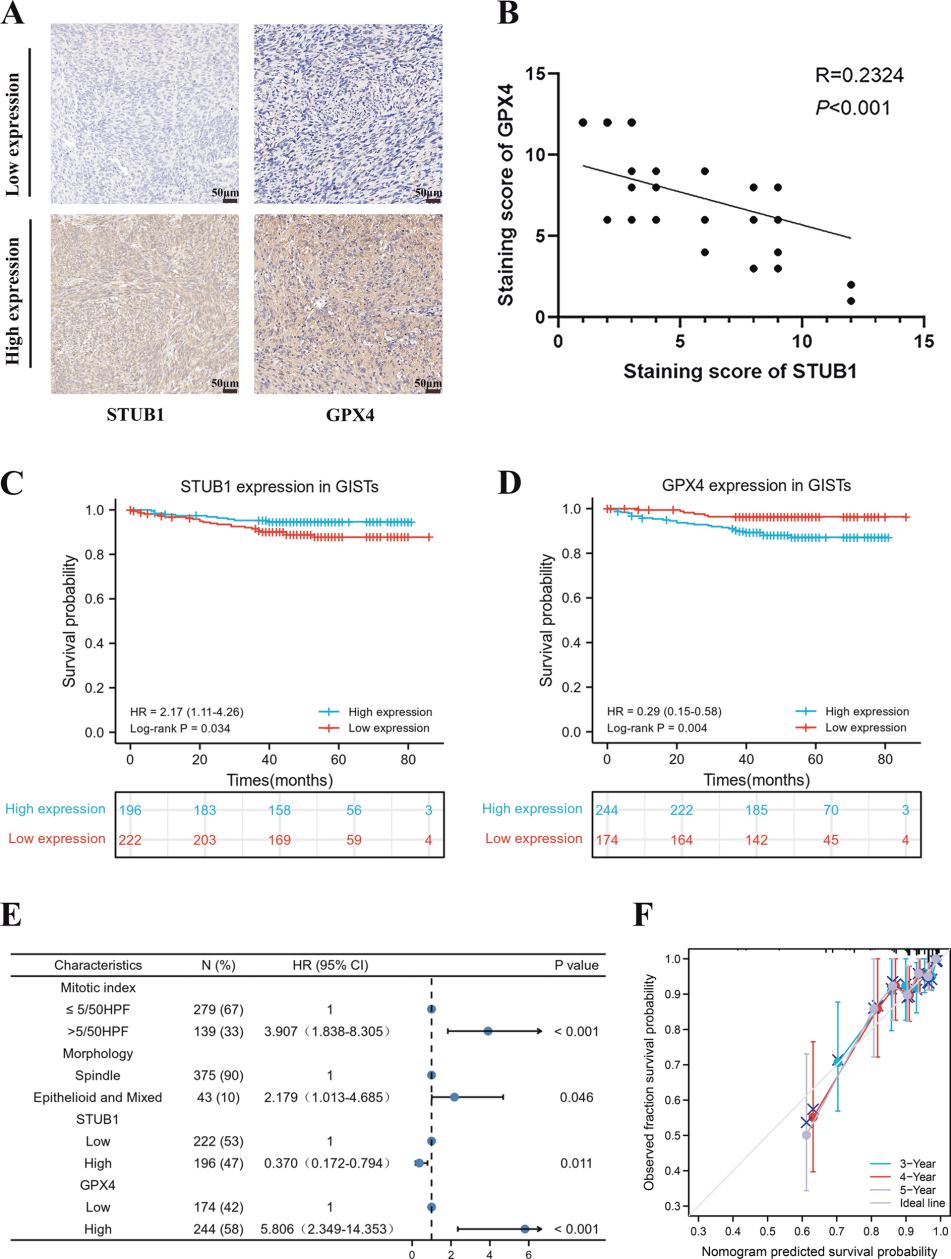
STUB1 and GPX4 are independent prognostic factors for gastrointestinal stromal tumor (GIST). **A** Immunohistochemical analysis for the STUB1 and GPX4 expression in GIST tissue microarrays (*n* = 418). **B** Scatter plot showing a negative correlation between the expression of STUB1 and GPX4 in GIST. **C** Low STUB1 expression predicted poor recurrence-free survival (RFS) in GIST patients. **D** High GPX4 expression predicted poor RFS in GIST patients. **E** Nomogram for predicting the probability of recurrence in patients with GIST. **F** Calibration plot of the nomogram. Scale bar, 50 μm in **A**.

Based on the staining intensity of STUB1 and GPX4 (Fig. 7A), IHC analysis of 418 tissue microarray samples revealed that there was a significant inverse correlation between the expression of STUB1 and GPX4 in GIST (Fig. 7B). Subsequently, correlations between STUB1 and GPX4 expression and clinicopathological features in the 418 patients with GIST were analyzed. The results showed that STUB1 expression was correlated with tumor location (*P* < 0.001), mitotic index (*P* < 0.001), NIH risk grade (*P* = 0.001), and cell morphology (*P* = 0.047). The expression of GPX4 was correlated with tumor location (*P* = 0.020), mitotic index (*P* = 0.019) and NIH risk grade (*P* = 0.005) (Table S4). The survival and recurrence status were last updated in August 2022. Among the 418 included patients, 397 patients (95.0%) were followed up completely and 21 were lost to follow-up. The median follow-up period was 48 (0–86) months. At the last follow-up, recurrence occurred in 34 cases (8.1%) and death in 4 (1.0%) cases. The overall 3- and 5-year recurrence-free survival(RFS) rate was 93.3% and 90.9% respectively. Log-rank analysis revealed significantly longer RFS in patients with high expression of STUB1 (*P* = 0.034) and low expression of GPX4 (*P* = 0.004; Fig. 7C, D). but there was no significant difference in overall survival(OS) (Fig. S9).

Univariate Cox analysis indicated that RFS was correlated with mitotic index (*P* < 0.001), cell morphology (*P* < 0.001), STUB1 expression (*P* = 0.039), and GPX4 expression (*P* = 0.006) (Table S5). The results of multivariate Cox analysis showed that mitotic index > 5/50HPF (HR: 3.907, 95%CI: 1.838–8.305, *P* < 0.001), epithelial and mixed cell type (HR: 2.179, 95%CI: 1.013–4.685, *P* = 0.046) and high GPX4 expression (HR: 5.806, 95%CI: 2.349–14.353, *P* < 0.001) were independent risk factors for recurrence (Table S5). The high expression of STUB1 (HR: 0.370, 95%CI: 0.172–0.794, *P* = 0.011) was an independent protective factor affecting the RFS of GIST patients. However, there was no correlation between OS and various factors (Table S6). Multivariate analysis was used to create a nomogram for survival prediction, and it was observed that compared to other clinical variables, STUB1 and GPX4 expression had a significant impact on RFS (Fig. 7E). The calibration plot showed good consistency between predicted and observed values, as the deviation correction line closely matched the ideal curve (45-degree line) (Fig. 7F). In summary, a valuable model for predicting recurrence in patients with GIST has been constructed.

## Discussion

The emergence of TKIs has improved the prognosis of GIST patients, however, approximately half of the patients develop acquired drug resistance due to secondary gene mutations within 2 years of treatment, which hinders the effectiveness of TKI treatment [28, 29]. Therefore, exploring new treatment strategies is imperative. Ferroptosis, a novel form of cell death has garnered significant attention [30, 31]. Eliminating tumors by targeting ferroptosis could be a potential therapeutic option for GIST. However, the role of ferroptosis in the tumorigenesis and progression of GIST and whether IM can induce ferroptosis remain unclear.

In this study, we first detected the presence of ferroptosis in GIST-T1 and GIST-882 cells and found that inhibition of ferroptosis by Ferrostatin-1 partially reversed the effect of IM on GIST, indicating that ferroptosis does exist in GIST. The detection indexes of ferroptosis include the changes in mitochondrial morphology and levels of lipid ROS, Fe^2+^, and GSH [32, 33]. We observed a reduction in mitochondrial volume and an increase in mitochondrial crests in GIST cells treated with IM. IM increased ROS and lipid ROS levels in GIST cells, and this effect could be reversed by Ferrostatin-1. In addition, IM promoted the accumulation of Fe^2+^ in GIST and decreased the GSH/GSSG ratio in a concentration-dependent manner. These results suggest that IM induces ferroptosis in GIST cells.

The mechanism of ferroptosis primarily involves classical pathways dependent on cysteine-glutamate antiporter (Systemxc-, is composed of SLC7A11 and SLC3A2) and GPX4 [34–36], and non-classical pathways such as abnormal lipid peroxide metabolism and iron metabolism [37, 38]. To clarify the specific molecular mechanism of IM-induced ferroptosis, we examined the effects of different IM concentrations and treatment durations on the expression of SLC7A11, GPX4, acyl-CoA synthetase long-chain family member 4 (ACSL4), ferritin-heavy polypeptide 1 (FTH1) and transferrin receptor 1 (TFR1) at the transcriptional level. Our results indicated no significant effect on the expression of ACSL4 (Fig. S10). The up-regulation of FTH1 and down-regulation of TFR1 may be caused by negative feedback (Fig. S10). Therefore, we hypothesize that the mechanism of ferroptosis caused by IM may be through the classical ferroptosis pathway mediated by GSH, and this speculation was later confirmed by the significant decrease of GSH/GSSG ratio after IM treatment. SLC7A11 and GPX4 are the two most critical molecules in the classical ferroptosis pathway [35, 39], in which no significant change was observed in the transcriptional and protein levels of SLC7A11, while GPX4 was significantly increased at the transcriptional level but significantly decreased at the protein level. These findings suggest that IM may regulate GPX4 expression through post-transcriptional modification. In addition, we found that IM increased the GPX4 mRNA levels, but this effect disappeared after adding proteasome inhibitor MG132, so we speculated that IM-induced GPX4 protein degradation may increase GPX4 mRNA levels through negative feedback (Figs. S11 and S12). Interestingly, we found that RSL3 could lead to inhibition of GPX4 transcripts as well (Fig. S11). This observation suggests that RSL3 might influence GPX4 at multiple levels, potentially leading to a complex regulatory response. One possibility is that RSL3, by inhibiting GPX4 activity and leading to lipid peroxidation, triggers a cellular stress response that results in alterations in gene expression, including GPX4 mRNA. It is also possible that RSL3 has indirect effects on GPX4 regulation, possibly through signaling pathways or transcription factors that control GPX4 expression. Further studies are needed to fully elucidate the mechanisms underlying the impact of RSL3 on GPX4 mRNA levels and to better understand the intricate regulatory networks involved in ferroptosis. The glutathione peroxidase protein family (GPXs) is an important protein for cells to resist peroxidation. GPX4 is the sole lipid peroxidation-reducing GPXs, capable of converting lipid peroxidation bonds into hydroxyl groups, thereby nullifying peroxidation activity and preventing the buildup of lipid peroxides. GPX4 serves as a key negative regulator of ferroptosis, with the loss of GPX4 enzyme activity being the primary cause of ferroptosis. Enhancing the inhibition of GPX4 expression or activity represents a novel strategy to augment ferroptosis effects.

As a widespread post-translational modification, ubiquitin plays a vital role in biological regulation [40, 41]. We further detected the ubiquitination level of GPX4 after IM treatment and found that IM could promote the ubiquitination of GPX4. Although the role of post-translational modification in ferroptosis has been gradually emphasized in recent years [42], it has been found that SOCS2-enhanced ubiquitination of SLC7A11 promotes ferroptosis in hepatocellular carcinoma [43], and ubiquitin-specific protease 7 (USP7) promotes ferroptosis via activation of the p53/TFR1 pathway [44], and NEDD4 ubiquitylates VDAC2/3 to suppress erastin-induced ferroptosis in melanoma [45]. However, the research on GPX4 ubiquitin is very limited [42]. To explore the specific molecules that mediate the ubiquitination of GPX4 induced by IM, we performed UbiBrowser website and found that STUB1 may be the E3 ubiquitin ligase of GPX4. Our subsequent DRUGSURV database analysis also showed that STUB1 may be the indirect target of IM, and that IM could upregulate STUB1 expression, and knockdown of STUB1 effectively blocked the ferroptosis induced by IM.

We subsequently investigated the specific mechanism by which STUB1 regulates GPX4. As a ubiquitin ligase, STUB1 functions by binding to substrates, promoting substrate degradation. Currently, HMGB1 [46], ErbB2 [47] and CIB1 [20] have been identified as the substrates of STUB1. Ubiquitin primarily regulates target proteins through covalent binding with substrate lysines. However, whether STUB1 regulates GPX4 through a similar lysine-related mechanism requires further investigation. In the present study, CO-IP results indicated that STUB1 inhibits GPX4 protein stability and promotes GPX4 ubiquitination through direct binding to GPX4. We constructed GPX4 deubiquitination point mutant plasmids (K107R, K126R, K162R, K167R, K191R). Immunoprecipitation results revealed that ubiquitin levels of GPX4 significantly decreased following deubiquitination mutation at K191, with concurrent inhibition of GPX4 ubiquitination mediated by STUB1. This suggests that K191 is a key regulatory site for GPX4 ubiquitin modification catalyzed by STUB1. Additionally, exploring more substrates could enhance our understanding of STUB1’s functions. Apart from the GSH transport system, ferroptosis is regulated by ferroptosis suppressor protein 1 (FSP1). FSP1 is a NAD(P)H-ubiquinone reductase, capable of reducing vitamin K to hydroquinone, a potent free radical trapping antioxidant, and an inhibitor of (phosphorus) lipid peroxidation. FSP1-dependent non-classical vitamin K cycling cooperates with GPX4 and glutathione to suppress lipid peroxidation and ferroptosis [48, 49]. Whether STUB1 can regulate FSP1 requires further exploration. In summary, our data suggest that IM in GIST promotes the ubiquitination at site K191 of GPX4 by promoting the expression of STUB1, resulting in the degradation of GPX4 protein and induction of ferroptosis.

Previous studies have demonstrated that the efficacy of IM depends on the type of gene mutation, with patients harboring c-kit exon 11 mutations being more sensitive to IM than those with PDGFRA exon 18 D842V mutations [50]. Our study highlights that IM could induce ferroptosis in GIST through GPX4, yet GPX4 expression is heterogeneous in patients with GIST. The expression of GPX4 was found to be negatively correlated with the prognosis of GIST. Generally, GIST patients with high expression of GPX4 may benefit more from IM treatment. Further elucidating the potential mechanism of STUB1 and GPX4 in GIST and screening new drug combinations based on IM could represent a promising strategy for the treatment of GIST in the future.

RSL3 is a specific inhibitor of GPX4 approved by the FDA, which has been shown to inhibit the growth, invasion, and metastasis of multiple cancers [51, 52]. Our data indicate that RSL3 and IM could synergistically inhibit the expression of GPX4 and promote ferroptosis both in vivo and in vitro. Consequently, RSL3 demonstrates effective anti-tumor activity against GIST, and when combined with IM, it may offer additional treatment options for patients with GIST with poor responses to IM.

However, it is important to acknowledge that our study has certain limitations. Specifically, the use of immunodeficient BALB/c nude mice may restrict the generalizability of our findings to the broader context of ferroptosis’s impact on the immune system. This encompasses aspects such as the immune tolerance of late-stage ferroptotic cancer cells and the immunogenicity of early-stage ferroptotic cancer cells.

## Conclusion

**Fig. 8:**
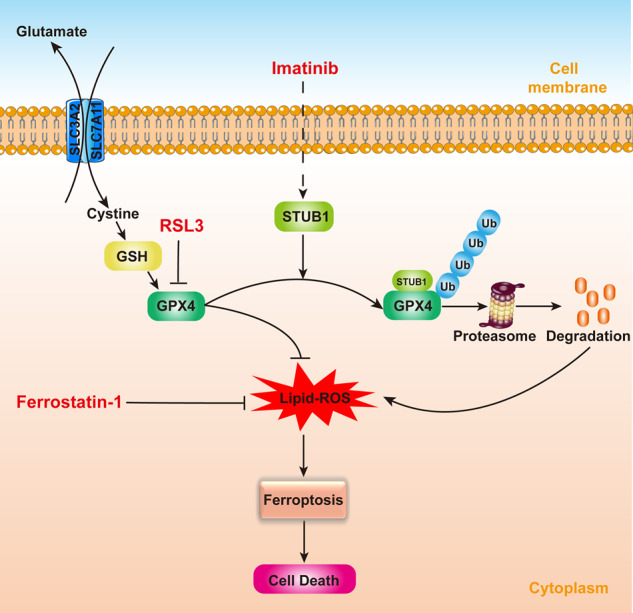
Mechanism diagram of imatinib (IM) inducing ferroptosis. IM upregulates STUB1 expression. STUB1 overexpression promotes the ubiquitination of GPX4, thereby inducing ferroptosis in gastrointestinal stromal tumors (GIST).

As pictorially modeled in Fig. 8, this study for the first time demonstrates that IM induces ferroptosis in GIST by promoting STUB1-mediated GPX4 ubiquitination, offering a novel mechanism for the inhibition of GIST. Furthermore, combining IM with RSL3 presents a promising therapeutic strategy for GIST.

### Supplementary information

Supplemental Material

AJ-checklist

## Data Availability

All data are available within the text and Supplementary Material.

## References

[1] Demetri GD, von Mehren M, Antonescu CR, DeMatteo RP, Ganjoo KN, Maki RG (2010). NCCN Task Force report: update on the management of patients with gastrointestinal stromal tumors. J Natl Compr Canc Netw.

[2] Heinrich MC, Corless CL, Duensing A, McGreevey L, Chen CJ, Joseph N (2003). PDGFRA activating mutations in gastrointestinal stromal tumors. Science.

[3] Demetri GD, von Mehren M, Blanke CD, Van den Abbeele AD, Eisenberg B, Roberts PJ (2002). Efficacy and safety of imatinib mesylate in advanced gastrointestinal stromal tumors. N Engl J Med.

[4] Kee D, Zalcberg JR (2012). Current and emerging strategies for the management of imatinib-refractory advanced gastrointestinal stromal tumors. Ther Adv Med Oncol.

[5] Klug LR, Khosroyani HM, Kent JD, Heinrich MC (2022). New treatment strategies for advanced-stage gastrointestinal stromal tumours. Nat Rev Clin Oncol.

[6] Dixon SJ, Lemberg KM, Lamprecht MR, Skouta R, Zaitsev EM, Gleason CE (2012). Ferroptosis: an iron-dependent form of nonapoptotic cell death. Cell.

[7] Liang D, Minikes AM, Jiang X (2022). Ferroptosis at the intersection of lipid metabolism and cellular signaling. Mol Cell.

[8] Cui S, Simmons GJ, Vale G, Deng Y, Kim J, Kim H (2022). FAF1 blocks ferroptosis by inhibiting peroxidation of polyunsaturated fatty acids. Proc Natl Acad Sci USA.

[9] Chen X, Li J, Kang R, Klionsky DJ, Tang D (2021). Ferroptosis: machinery and regulation. Autophagy.

[10] Seibt TM, Proneth B, Conrad M (2019). Role of GPX4 in ferroptosis and its pharmacological implication. Free Radic Biol Med.

[11] Xu T, Ding W, Ji X, Ao X, Liu Y, Yu W (2019). Molecular mechanisms of ferroptosis and its role in cancer therapy. J Cell Mol Med.

[12] Li Y, Zeng X, Lu D, Yin M, Shan M, Gao Y (2021). Erastin induces ferroptosis via ferroportin-mediated iron accumulation in endometriosis. Hum Reprod.

[13] Zhuang J, Liu X, Yang Y, Zhang Y, Guan G (2021). Sulfasalazine, a potent suppressor of gastric cancer proliferation and metastasis by inhibition of xCT: conventional drug in new use. J Cell Mol Med.

[14] Zhang X, Guo Y, Li H, Han L (2021). FIN56, a novel ferroptosis inducer, triggers lysosomal membrane permeabilization in a TFEB-dependent manner in glioblastoma. J Cancer.

[15] Sun Y, Berleth N, Wu W, Schlutermann D, Deitersen J, Stuhldreier F (2021). Fin56-induced ferroptosis is supported by autophagy-mediated GPX4 degradation and functions synergistically with mTOR inhibition to kill bladder cancer cells. Cell Death Dis.

[16] Chen X, Kang R, Kroemer G, Tang D (2021). Broadening horizons: the role of ferroptosis in cancer. Nat Rev Clin Oncol.

[17] Yang J, Zhou Y, Xie S, Wang J, Li Z, Chen L (2021). Metformin induces Ferroptosis by inhibiting UFMylation of SLC7A11 in breast cancer. J Exp Clin Cancer Res.

[18] Liu C, Lou W, Yang JC, Liu L, Armstrong CM, Lombard AP (2018). Proteostasis by STUB1/HSP70 complex controls sensitivity to androgen receptor targeted therapy in advanced prostate cancer. Nat Commun.

[19] Shi CH, Rubel C, Soss SE, Sanchez-Hodge R, Zhang S, Madrigal SC (2018). Disrupted structure and aberrant function of CHIP mediates the loss of motor and cognitive function in preclinical models of SCAR16. PLoS Genet.

[20] Liu Y, Zhou Y, Zhang P, Li X, Duan C, Zhang C (2021). CHIP-mediated CIB1 ubiquitination regulated epithelial-mesenchymal transition and tumor metastasis in lung adenocarcinoma. Cell Death Differ.

[21] Paul I, Ahmed SF, Bhowmik A, Deb S, Ghosh MK (2013). The ubiquitin ligase CHIP regulates c-Myc stability and transcriptional activity. Oncogene.

[22] Ahmed SF, Deb S, Paul I, Chatterjee A, Mandal T, Chatterjee U (2012). The chaperone-assisted E3 ligase C terminus of Hsc70-interacting protein (CHIP) targets PTEN for proteasomal degradation. J Biol Chem.

[23] Stauffer W, Sheng H, Lim HN (2018). EzColocalization: an ImageJ plugin for visualizing and measuring colocalization in cells and organisms. Sci Rep.

[24] Ishida T, Takahashi T, Kurokawa Y, Nishida T, Hirota S, Serada S (2021). Targeted therapy for drug-tolerant persister cells after imatinib treatment for gastrointestinal stromal tumours. Br J Cancer.

[25] Liu J, Gao J, Wang A, Jiang Z, Qi S, Qi Z (2022). Nintedanib overcomes drug resistance from upregulation of FGFR signalling and imatinib-induced KIT mutations in gastrointestinal stromal tumours. Mol Oncol.

[26] Amelio I, Gostev M, Knight RA, Willis AE, Melino G, Antonov AV (2014). DRUGSURV: a resource for repositioning of approved and experimental drugs in oncology based on patient survival information. Cell Death Dis.

[27] Chou TC (2010). Drug combination studies and their synergy quantification using the Chou-Talalay method. Cancer Res.

[28] Zhao Q, Zhang C, Qi C, Yang J, Chen Y, Ge S (2021). Preclinical model-based evaluation of Imatinib resistance induced by KIT mutations and its overcoming strategies in gastrointestinal stromal tumor (GIST). Am J Transl Res.

[29] Agulnik M, Giel JL (2014). Understanding rechallenge and resistance in the tyrosine kinase inhibitor era: imatinib in gastrointestinal stromal tumor. Am J Clin Oncol.

[30] Lei G, Zhang Y, Koppula P, Liu X, Zhang J, Lin SH (2020). The role of ferroptosis in ionizing radiation-induced cell death and tumor suppression. Cell Res.

[31] Mbah NE, Lyssiotis CA (2022). Metabolic regulation of ferroptosis in the tumor microenvironment. J Biol Chem.

[32] Zeng F, Nijiati S, Tang L, Ye J, Zhou Z, Chen X (2023). Ferroptosis detection: from approaches to applications. Angew Chem Int Ed Engl.

[33] Zheng H, Jiang J, Xu S, Liu W, Xie Q, Cai X (2021). Nanoparticle-induced ferroptosis: detection methods, mechanisms and applications. Nanoscale.

[34] Conrad M, Kagan VE, Bayir H, Pagnussat GC, Head B, Traber MG (2018). Regulation of lipid peroxidation and ferroptosis in diverse species. Genes Dev.

[35] Zhang X, Zheng C, Gao Z, Chen H, Li K, Wang L (2022). SLC7A11/xCT prevents cardiac hypertrophy by inhibiting ferroptosis. Cardiovasc Drugs Ther.

[36] Yang WS, SriRamaratnam R, Welsch ME, Shimada K, Skouta R, Viswanathan VS (2014). Regulation of ferroptotic cancer cell death by GPX4. Cell.

[37] Wang S, Wei W, Ma N, Qu Y, Liu Q (2022). Molecular mechanisms of ferroptosis and its role in prostate cancer therapy. Crit Rev Oncol Hematol.

[38] Liu J, Kang R, Tang D (2022). Signaling pathways and defense mechanisms of ferroptosis. FEBS J.

[39] Forcina GC, Dixon SJ (2019). GPX4 at the crossroads of lipid homeostasis and ferroptosis. Proteomics.

[40] Hwang JT, Lee A, Kho C (2022). Ubiquitin and ubiquitin-like proteins in cancer, neurodegenerative disorders, and heart diseases. Int J Mol Sci.

[41] Husnjak K, Dikic I (2012). Ubiquitin-binding proteins: decoders of ubiquitin-mediated cellular functions. Annu Rev Biochem.

[42] Meng Y, Sun H, Li Y, Zhao S, Su J, Zeng F (2022). Targeting ferroptosis by ubiquitin system enzymes: a potential therapeutic strategy in cancer. Int J Biol Sci.

[43] Chen Q, Zheng W, Guan J, Liu H, Dan Y, Zhu L (2023). SOCS2-enhanced ubiquitination of SLC7A11 promotes ferroptosis and radiosensitization in hepatocellular carcinoma. Cell Death Differ.

[44] Tang LJ, Zhou YJ, Xiong XM, Li NS, Zhang JJ, Luo XJ (2021). Ubiquitin-specific protease 7 promotes ferroptosis via activation of the p53/TfR1 pathway in the rat hearts after ischemia/reperfusion. Free Radic Biol Med.

[45] Yang Y, Luo M, Zhang K, Zhang J, Gao T, Connell DO (2020). Nedd4 ubiquitylates VDAC2/3 to suppress erastin-induced ferroptosis in melanoma. Nat Commun.

[46] Sun Y, Wang Q, Wang M, Sun F, Qiao P, Jiang A (2022). CHIP induces ubiquitination and degradation of HMGB1 to regulate glycolysis in ovarian endometriosis. Cell Mol Life Sci.

[47] Luan H, Bailey TA, Clubb RJ, Mohapatra BC, Bhat AM, Chakraborty S (2021). CHIP/STUB1 ubiquitin ligase functions as a negative regulator of ErbB2 by promoting its early post-biosynthesis degradation. Cancers.

[48] Doll S, Freitas FP, Shah R, Aldrovandi M, Da SM, Ingold I (2019). FSP1 is a glutathione-independent ferroptosis suppressor. Nature.

[49] Bersuker K, Hendricks JM, Li Z, Magtanong L, Ford B, Tang PH (2019). The CoQ oxidoreductase FSP1 acts parallel to GPX4 to inhibit ferroptosis. Nature.

[50] Antonescu CR, Besmer P, Guo T, Arkun K, Hom G, Koryotowski B (2005). Acquired resistance to imatinib in gastrointestinal stromal tumor occurs through secondary gene mutation. Clin Cancer Res.

[51] Wang C, Zheng C, Wang H, Shui S, Jin H, Liu G (2023). Dual degradation mechanism of GPX4 degrader in induction of ferroptosis exerting anti-resistant tumor effect. Eur J Med Chem.

[52] Sui X, Zhang R, Liu S, Duan T, Zhai L, Zhang M (2018). RSL3 drives ferroptosis through GPX4 inactivation and ROS production in colorectal cancer. Front Pharmacol.

