# Supplementary material for: Imatinib induces ferroptosis in gastrointestinal stromal tumors by promoting STUB1-mediated GPX4 ubiquitination
Source: Cell Death Dis. 2023 Dec 18;14(12):839. doi: 10.1038/s41419-023-06300-2 (PMC10728200; doi:10.1038/s41419-023-06300-2)
Supplement: Supplementary file 1 — Supplemental Material [file 41419_2023_6300_MOESM1_ESM.pdf]

Supplementary Material

Imatinib induces ferroptosis in gastrointestinal stromal tumors by promoting STUB1-mediated GPX4 ubiquitination

Xiangfei Sun, Qiang Zhang, Xiaohan Lin, Ping Shu, Xiaodong Gao, Kuntang Shen

Supplementary Figure 1-12

Supplementary Table 1-6

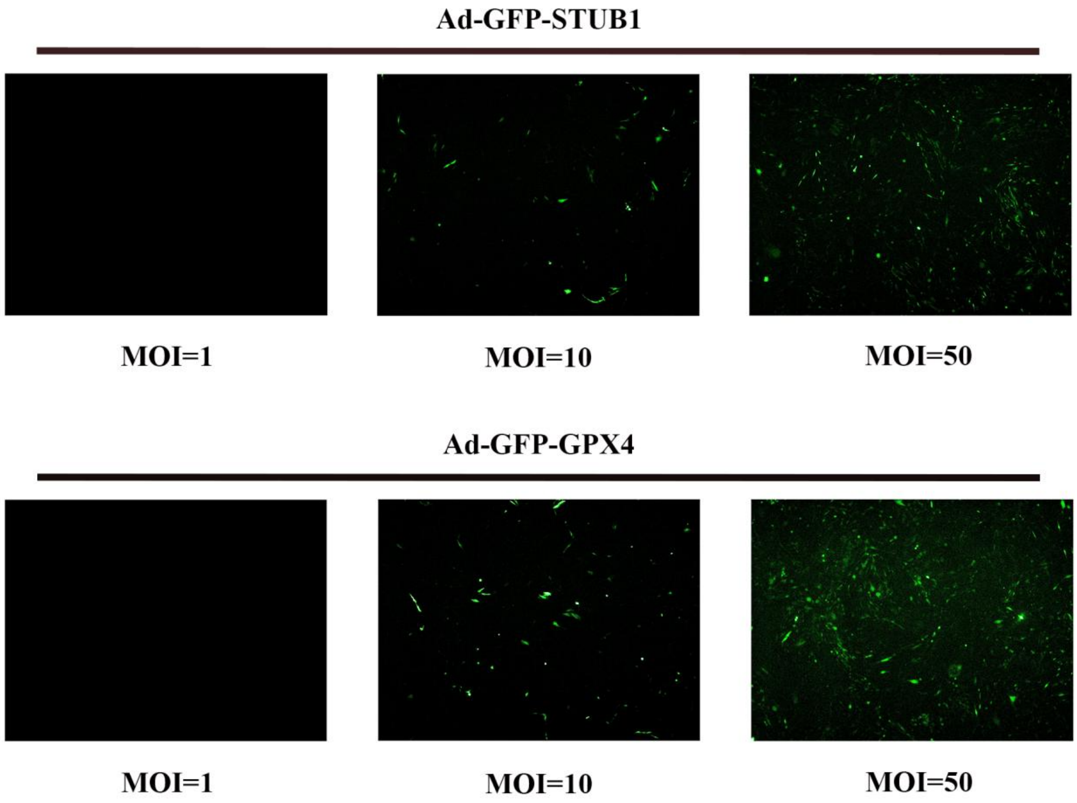

Supplementary Figure 1. Adenovirus expression efficiency on STUB1 and GPX4 in GIST-T1.

Adenovirus encoding green fluorescent protein was detected 72 h after the adenovirus transfection in GIST-T1.

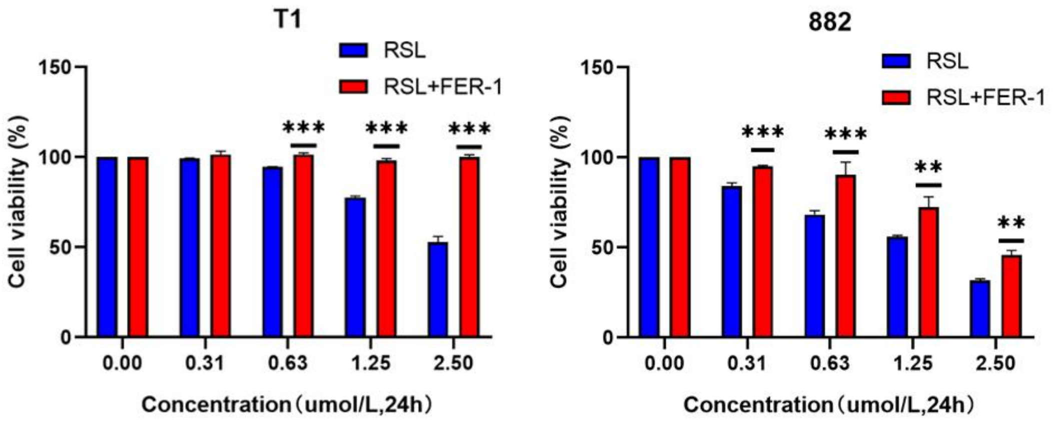

Supplementary Figure 2. GIST-T1 and 882 cells were treated with RSL3 (0, 0.31, 0.63, 1.25 and 2.50 μM) for 24 h in the absence or presence of Ferrostatin-1 (FER-1) (1 uM), and cell viability was assayed.

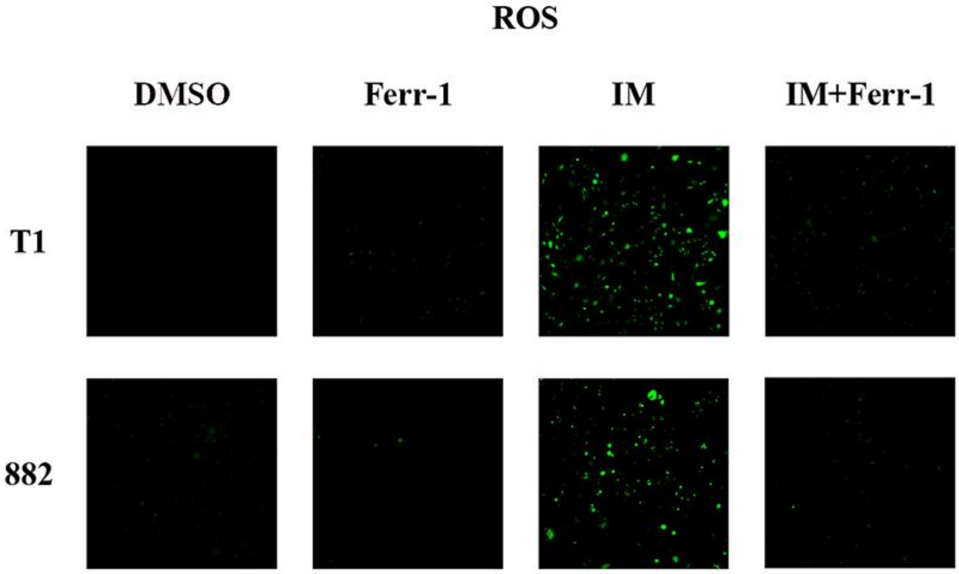

Supplementary Figure 3. GIST-T1 and 882 cells were treated with IM (50 nM for T1 and 200 nM for 882) for 12 h, and the relative ROS levels were assayed via DCFH-DA fluorescence.

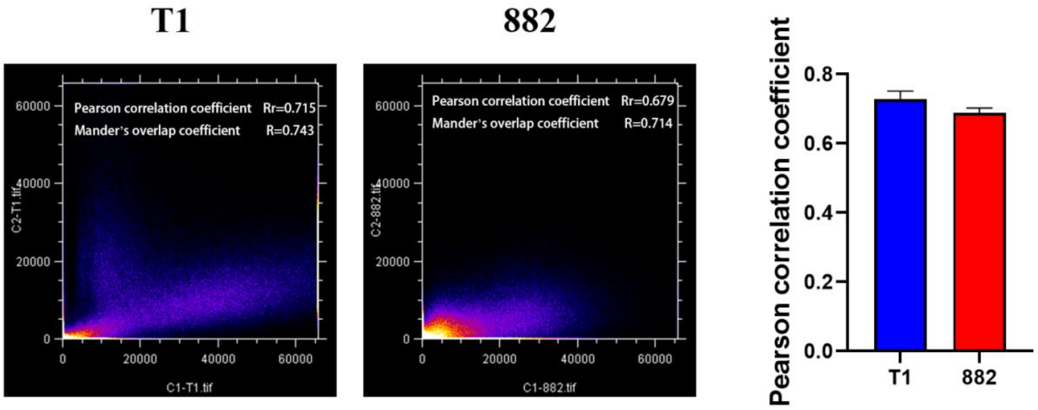

Supplementary Figure 4. Pearson correlation coefficient for localization of STUB1 and GPX4 in GIST-T1 and 882 cells by ImageJ software with the Colocalization Finder plugin.

There was co-localization of STUB1 and GPX4 in the cytoplasm of both GIST-T1 (Pearson correlation coefficient = 0.728 ± 0.024) and GIST-882 cells (Pearson correlation coefficient = 0.689 ± 0.013). Pearson correlation coefficient (>0.5) describes the correlation of the intensity distribution between channels. Manders' overlap coefficient (>0.6) indicates an actual overlap of the signals and is considered to represent the true degree of colocalization.

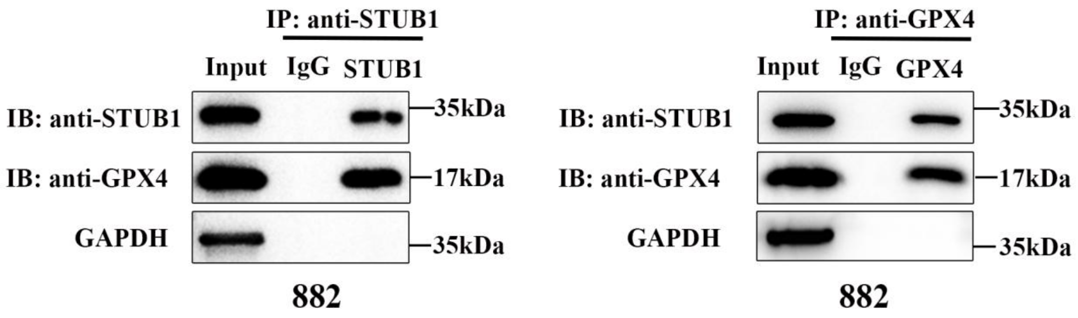

Supplementary Figure 5. Direct interaction between STUB1 and GPX4 in GIST-882 cells. Protein-protein interactions in GIST-882 cells were validated by a CO-IP assay using an anti-STUB1 and an anti-GPX4 antibody. An antibody targeting IgG was used as the negative control.

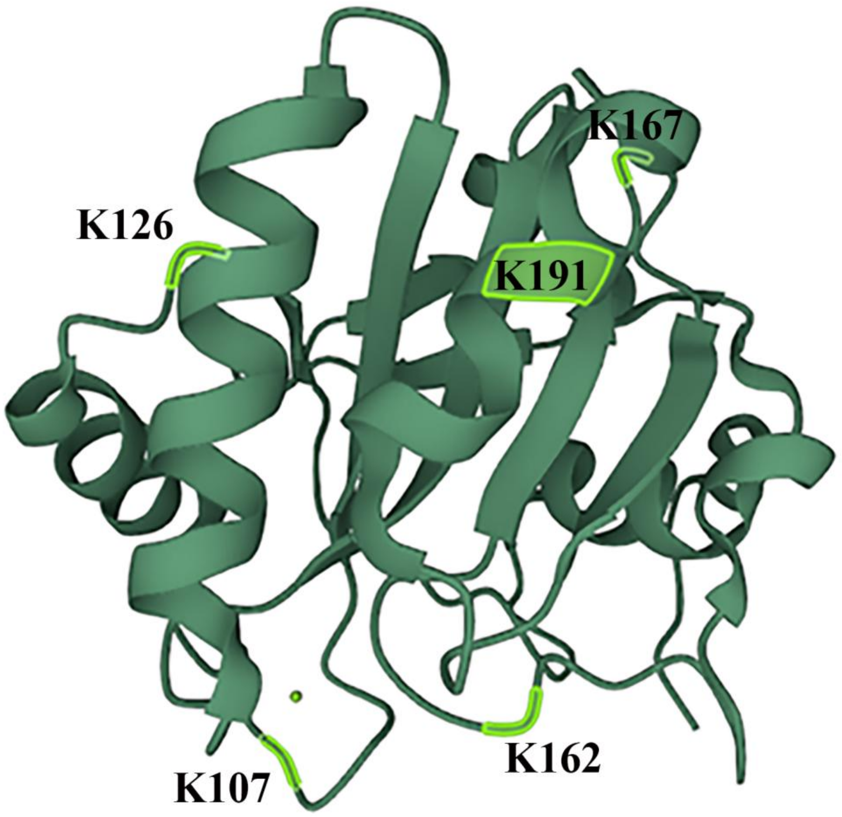

Supplementary Figure 6. Spatial structure of lysine sites of ubiquitination of GPX4.

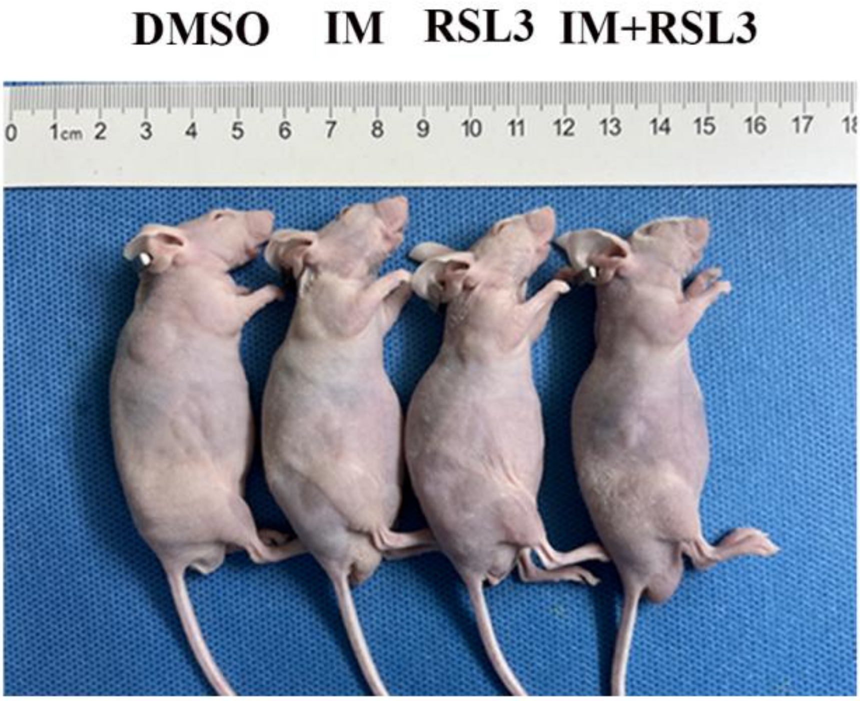

Supplementary Figure 7. Representative mouse images of each treatment group on day 18 after treatment, including vehicle (DMSO), IM (100 mg/kg), RSL3 (10 mg/kg) or IM combined with RSL3 treatment groups.

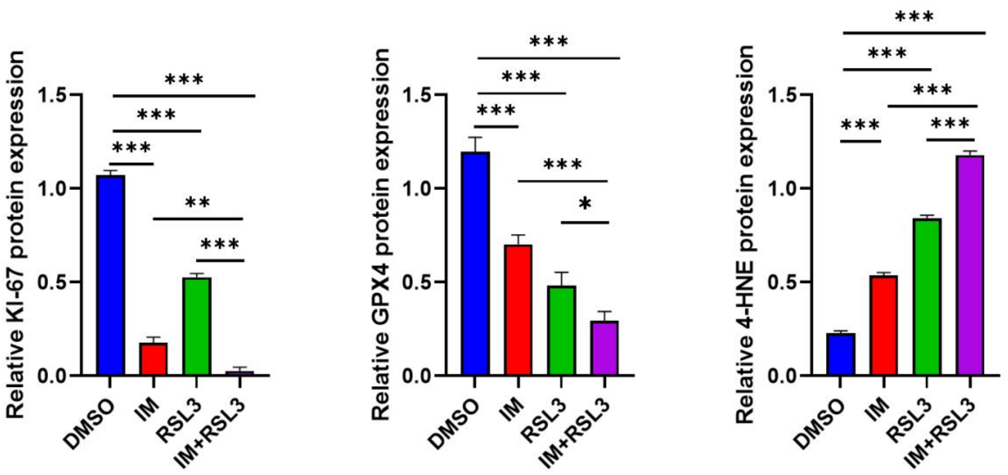

Supplementary Figure 8. Quantitative analysis of Ki-67, GPX4, and 4-HNE protein levels in GIST cell xenograft tumor tissues of mice from vehicle (DMSO), IM, RSL3 and combination treatment groups. n = 3 mice per group.

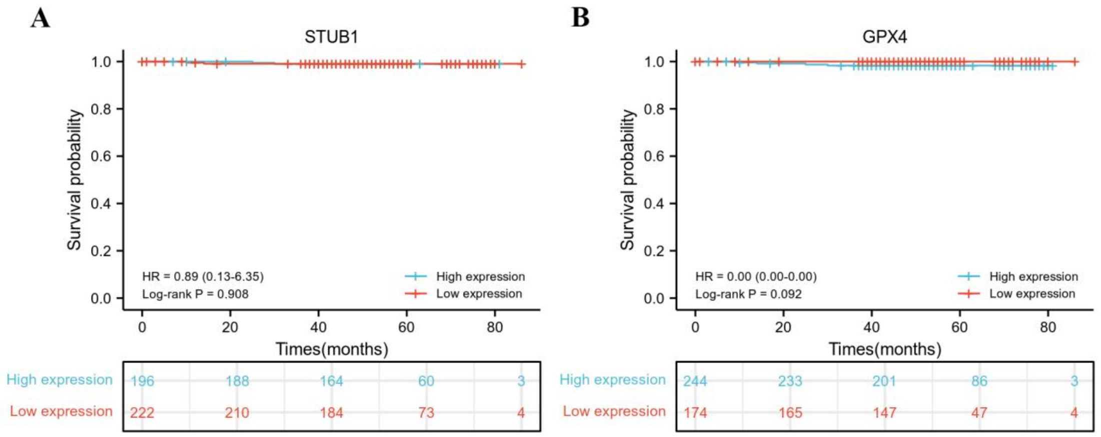

Supplementary Figure 9. Kaplan-Meier analysis of the relationships between high and low expression of STUB1 and GPX4 and overall survival in gastrointestinal stromal tumors (GIST).

A There was no statistical difference in OS between patients with high and low expression of STUB1(log-rank, P = 0.908). **B** There was no statistical difference in OS between patients with high and low expression GPX4 (log-rank, P = 0.092).

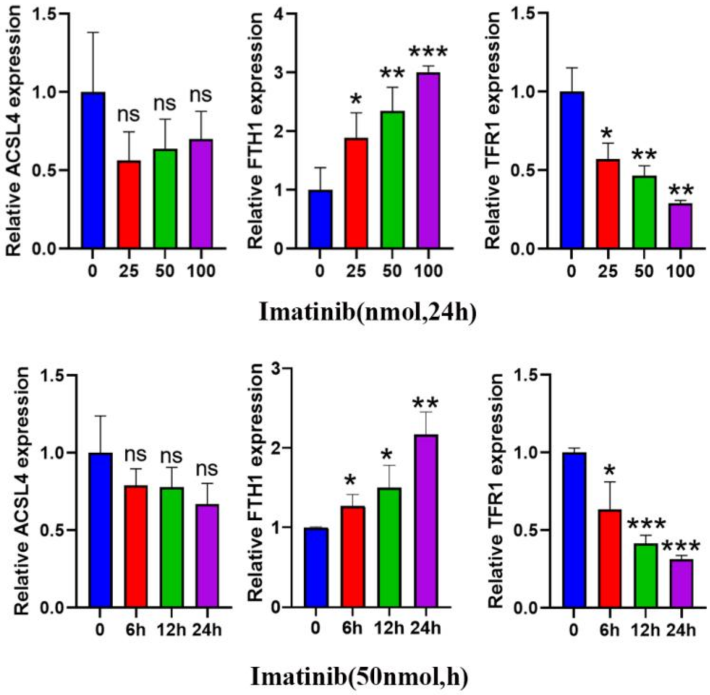

Supplementary Figure 10. The relative ASCL4, FTH1 and TFR1 RNA level was measured via qRT-PCR after GIST-T1 cells were treated with IM at 0, 25, 50, or 100 nM for 24 h or treated with IM (50 nM) for 0, 6, 12, or 24 h.

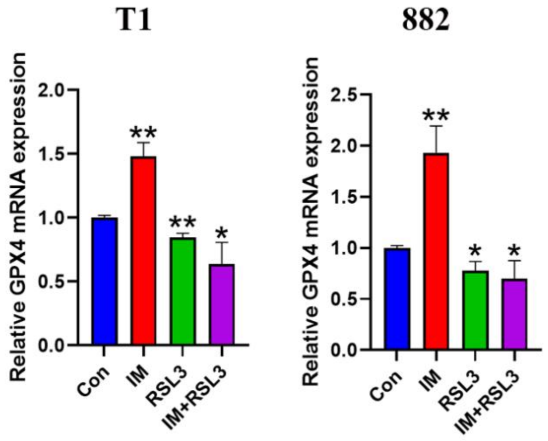

Supplementary Figure 11. The relative GPX4 mRNA level was measured via qRT-PCR after GIST-T1 and 882 cells were treated with IM (50 nM for T1 and 200 nM for 882) and RSL3 (2 μM for T1 and 1 μM for 882) for 24 h. Significance denoted by: ns, not significant, *P<0.05, **P<0.01, and ***P<0.001.

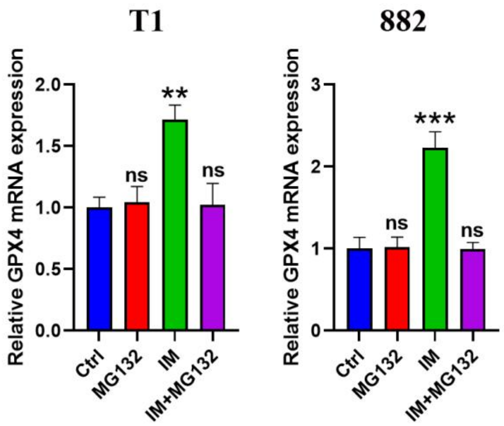

Supplementary Figure 12. The relative GPX4 mRNA levels were measured via qRT-PCR after GIST-T1 and 882 cells were treated with IM (50 nM for T1 and 200 nM for 882) in the absence or presence of MG132 (5 μM) for 24 h. Significance denoted by: ns, not significant, *P<0.05, **P<0.01, and ***P<0.001.

Supplementary Table 1. Sequence of qRT-PCR primers.

Gene name	Forward(5'-3')	Reverse(5'-3')
SLC7A11	TCCGCAAGCACACTCCTCTACC	GTGATGACGAAGCCAATCCCTGTAC
GPX4	CCCGATACGCTGAGTGTGGTTTG	CCTTGCCCTTGGGTTGGATCTTC
FTH1	GGAGAGGGAACATGCTGAGAAACTG	AGATATTCCGCCAAGCCAGATTCG
TFR1	GCTGTATTCTGCTCGTGGAGACTTC	CGTCACCAGAGAGGGCATTTGC
ACSL4	TGGGCTAAATGAATCTGAGGCTTCC	GGCGTTGGTCTACTTGGAGGAATG
GAPDH	CAAGGCTGTGGGCAAGGTCATC	GTGTCGCTGTTGAAGTCAGAGGAG
STUB1	AAGCGAGACATCCCCGACTACC	TGCGTCAATAACCTCCTTCATAGCC

Supplementary Table 2. Antibodies used in this study.

Antibody	Company	Product number	Dilution	Application
GAPDH	Abcam	ab9485	1:2000	WB
SLC7A11	Abcam	ab37185	1:1000	WB
STUB1	Abcam	ab134064	1:10000	WB
			1:250	IF
			1:100	IHC
				IP
GPX4	Abcam	ab125066	1:1000	WB
			1:100	IHC
			1:200	IF
	Proteintech	67763-1-Ig		IP
			1:200	IF
Anti-Ubiquitin	Abcam	ab134953	1:1000	WB
4-HNE	Invitrogen	MA5-27570	1:50	IHC
			1:1000	WB
Ki-67	Servicebio	GB121141	1:300	IHC
	Abcam	ab46545	1:1000	WB
KIT	Servicebio	GB11073-2	1:400	IHC
	Abcam	ab32363	1:1000	WB
Rabbit (DA1E) mAb IgG	Cell Signaling Technology	3900		IP
Anti-mouse IgG, HRP-linked Antibody	Cell Signaling Technology	7076	1:1000	WB
Anti-rabbit IgG, HRP-linked Antibody	Cell Signaling Technology	7074	1:1000	WB

Supplementary Table 3. Predicted E3 ubiquitin ligase of GPX4 and predicted direct and indirect targets of Imatinib (IM).

E3 ubiquitin ligase	Direct or indirect targets of Imatinib					E3 ubiquitin ligase AND targets of Imatinib
MIB2	ABL1	DAB2	SORBS2	IKBKG	C20ORF3	STUB1
NEDD4L	ABL2	CSNK2A1	SSU72	EEF1G	TULP1	CBL
MDM2	BLK	ARRB1	PSMC5	CHUK	PJA1	HSPA8
UBE4B	BRAF	TDP2	CDK4	DNM3	RRAS	
UBE4A	CA1	ERBB4	DOCK1	CAST	HSPA1B	
NEDD4	CA12	ERBB3	PTPN22	PHACTR2	PPP5C	
PAFAH1B1	CA14	STAM2	RRAS2	SLC5A1	APOL5	
M ARCH9	CA2	ID2	DAG1	AHSG	EIF3D	
SYVN1	CA3	SYNJ2	LAT	PXN	ATIC	
MIB1	CA4	F2RL2	ATP5B	DNAJB4	KIAA1712	
FZR1	CA5A	HSPA4	PLG	SP1	PTPRZ1	
SMURF1	CA5B	MED16	HIST1H3H	PALM2	MYCBPAP	
PPIL2	CA6	MED10	KDR	ALDOA	G3BP2	
BMI1	CA7	HSP90AA1	S100A11	KRT10	PRX	
RANBP2	CA9	DCAF7	RPP38	MSH5	PTPRG	
ZMIZ1	CDK19	WWP2	SREBF2	SCARF2	CNTFR	
CRYAB	CLK1	EPB41L3	SNRNP40	PDGFB	AIRE	
PML	CLK4	MAP4K5	CD22	CSRP1	DNAJA1	
ZEB2	CSF1R	RTN4	CEND1	EPHA2	CCR10	
TRIM33	CYP2C19	HRAS	MED23	MAP3K10	NGF	
UBE3C	CYP2C9	TGOLN2	KITLG	SUV39H2	LGALS7	
TTC3	CYP2D6	SLC24A1	TDRD1	SMAD4	TTR	
TRIM2	CYP3A4	FAM46C	PTPN1	SLC25A12	MEPE	
RNF216	CYP3A5	HEXIM1	SHC1	TNS3	UNC119	
	CYP3A7	YWHAB	GLTSCR1	PKD1	HSD17B7	
	DDR1	CAV1	STIP1	MED17	PIN1	
	DDR2	TM4SF1	DLGAP4	ZC3H12A	DNAJA2	
	EGFR	PECAM1	NBEA	PTPRK	DNM1	
	EPHA8	TUSC2	GAB2	KIFC2	MAP4K1	
	FGR	IRS1	TSC22D4	DLGAP1	ST5	
	FLT3	TXN	SF3B4	WNK2	HOXC8	

	FRK	TAX1BP3	MAP3K7	SH2D2A	RIN1	
	FYN	SMAD2	AK4	HEXA	HCN4	
	GAK	CDKN1B	LRBA	GPR45	SLC25A3	
	HIPK4	FASLG	MED24	RAPGEF1	HIST1H2BE	
	HRH2	LIG3	VPS13A	HIST1H2BF	C19ORF62	
	HTR2A	FYB	MDH1	PRDX2	LGALS7B	
	IRAK1	MTUS2	PHKB	NR1I2	CNTNAP1	
	KIT	SOS2	MED8	RDH13	RIMS2	
	LCK	RPL4	PRKAR1B	HSPB1	PELI2	
	LYN	C11ORF57	TIMM50	PRCC	FHL2	
	MAPK10	MKNK2	CRK	TCAP	HIST1H3B	
	MAPK8	PELI1	HSPA1A	PAEP	RIMS1	
	MAPK9	PTEN	MAP3K12	PRKCD	NUDT14	
	MELK	BMP8B	HP	HIST1H2BI	CAMP	
	NTRK1	HSPD1	PCK2	CBLB	UBB	
	PDGFRA	CTNNA1	HADHB	DLX4	BCL2L14	
	PDGFRB	CDC27	PTPRJ	SCOC	MED28	
	PIP4K2C	PLCH1	ISL1	DNASE1L2	ZAP70	
	PLK4	CYTH2	MAST1	KIF26A	ALS2CR12	
	RAF1	FKBP4	POLD1	TRIM39	CD2AP	
	RET	ATP5C1	ARPC5	SKI	FABP3	
	SLC6A4	ARAF	ARF6	TRAF6	RAB11FIP5	
	SMAD3	TBRG4	GUCY1A3	SMURF2	CALM2	
	SRC	C1ORF116	TRAIP	CLN3	XRCC1	
	STK17A	CSMD2	CDH1	MED12	HIST1H3C	
	TBXAS1	CFH	PDIA2	STK16	HOXC10	
	TNNI3K	KPNB1	HCN2	HIST1H3F	DHX8	
	TYK2	YWHAE	CHRD	PIH1D1	MED11	
	ZAK	TUBB	BCAR3	SRY	AATF	
	MKI67	CCT3	HSPA5	PTPRE	HIST1H3I	
	CENPA	TUBA1A	SNX17	BCAR1	LMAN2	
	CSE1L	KRT6A	IL3RA	SEPN1	DLGAP2	
	GAPDH	PIN4	EPHB4	RELA	RNF181	
	GSN	ACP5	HAX1	RAB3A	MYBPC3	
	FN1	DCTN2	PTK2B	PPP1R3D	MED26	
	FBLN1	AGL	SNX8	PLCG1	HIST1H3J	
	CLU	AIP	MED30	C6	CDC37	
	PTGDS	RIN3	COBRA1	TPD52L1	NEK8	
	IKBKB	BAZ2A	BEND5	KRTAP4-12	AXIN1	
	TPT1	JUN	PRICKLE3	BRCA2	RCVRN	
	PIK3R1	TGFBR2	MED21	SNAP25	GLTPD1	

	TMPO	SNX3	ETV5	CSK	GP6	
	HIST1H2BG	PKP2	MED19	GRIP2	DKKL1	
	CDK1	DACH1	ZP2	WDR62	HIVEP1	
	ACTN1	YWHAH	ATOH8	HNRNPH1	KRT5	
	ACTB	PPP1R12C	SNAPIN	MAPK8IP1	GUCY2D	
	GARS	ID4	VAV2	GRB2	FAM83G	
	CDC25A	NKX2-1	MOS	TULP4	MED29	
	TCP1	USP7	PTCHD2	SLC3A2	PHLDA3	
	TPI1	UCHL1	SQSTM1	PIAS4	S100A9	
	PSMA7	GTF3A	GSTZ1	IRAK4		
	ADRM1	YY1	MED18	ABR		
	CKAP4	HSD17B4	ACTA1	YWHAG		
	OIP5	AR	RB1	PTPRR		
	FOXO3	HNRNPR	NR4A1	SNW1		
	PLIN2	SH3BGRL	CCNT1	GPR63		
	FANCA	SGSM2	FOXF2	GABARAPL2		
	MATR3	YWHAQ	C2ORF3	RB1CC1		
	MACF1	FKBP8	NR5A1	MED31		
	ALCAM	SORBS1	MED20	RIOK2		
	PBK	CLTA	RNF32	COPS3		
	CALM1	MED27	RAB3D	CDKL5		
	PTPRB	OGN	ATXN1	ACTC1		
	TANK	NCK1	ZCCHC14	PPP3CA		
	ATP2A2	PARP4	UBL5	TNNI3		
	SEPP1	HDAC6	FAH	ACAP1		
	CUL7	HIST1H2BD	BRPF3	C4ORF49		
	ROR1	SPHK2	COL9A3	ZNF567		
	EEF1A1	PPP1R12A	PPP2CB	HIST1H3A		
	ORC1	CLTCL1	AP2A1	HIST1H3G		
	ISG20L2	CELSR2	GAD1	AKAP6		
	CAV2	JAK2	GOLM1	DOCK3		
	DYNC1H1	RAP1GDS1	GNS	FAM110A		
	PSMD4	SPCS2	FLNC	C20ORF46		
	YWHAZ	TOE1	PRDX1	TNIP1		
	IQGAP1	FBXO38	NDN	EFNB1		
	PRKCQ	ATP1B1	ITGB1	KRT1		
	ASAP1	TAB1	LEMD3	SRCIN1		
	DNAJC4	PTPN12	CDK8	RNF31		
	ITGB2	CELSR3	SEC13	NPVF		
	CFLAR	PEX19	ERRFI1	INTS10		
	CD59	PTPRC	XPOT	UBE2F		

	XPNPEP1	FLNA	ASB16	PAX7		
	RPLP2	RPA1	PTK2	HNRNPF		
	AHNAK	UBQLN4	TUBA4A	CBLL1		
	KRT14	ANXA1	LBP	RAP1A		
	PIK3C2A	GOT1	RALGAPB	SH3BGRL3		
	CHAF1A	HSPA9	PTPN4	OSBPL5		
	EPS15	ARHGAP33	STAT3	MED7		
	EGF	CUX1	EFNA5	ARHGEF11		
	MAPK1	TLR4	AKAP2	ITGA4		
	XPO7	MYC	EIF2B3	PTPN6		
	FAS	ARHGAP32	UROD	CFL1		
	MOBKL3	KIAA1377	CYP4F2	PSPC1		
	ULBP2	MAP3K3	NCKIPSD	PIK3C2B		
	STAMBP	NFASC	HTR6	TNFRSF13B		
	CCDC88A	AKAP12	PALM2-A<br>KAP2	GSX2		
	LRRC59	LTBP2	ANKZF1	C1ORF113		
	RGS20	DNM2	USP42	SERTAD1		
	UBE2V2	MLL4	ATP1A1	IL1F7		
	CYHR1	TERF1	NOTCH3	MED6		
	ATM	DOCK9	SLC22A3	JPH1		
	G3BP1	MED13	WASL	IRAK2		
	CREBBP	GK	C15ORF29	DLGAP3		
	RPL13	CDCP1	CALM3	MED4		
	MED13L	GTF2IRD1	HIST1H1E	CORO1A		
	ERBB2	TSKS	LIMK1	NXPH3		
	WIPF1	GANAB	HNRNPK	CACNA1F		
	PTPRM	CEP290	CTAGE5	MYD88		
	RBBP8	KCTD9	MED9	MED15		
	ITGB3	DNAJA3	GAB1	UHRF2		
	PNPLA2	RGS2	RPS6KB1	SYN1		
	TNK2	MAP2K2	CUL9	SEMA7A		
	ZMYND11	MEGF6	AHSA1	SHANK3		
	RPL3	ZFYVE9	MAP2K1	APOB		
	LRP2	IRAK3	PROX1	HCK		
	PAK2	AP2M1	ABI1	IL1F5		
	GPM6B	OLFM1	MET	PRTN3		
	SPHK1	PAX3	TRIM29	CD97		
	UHMK1	STAT5A	RPS6KA5	CNTN2		
	CKAP5	CSNK2B	HIST1H2B<br>C	VPREB1		
	SHANK2	CD247	PDK3	RUSC2		
	GTF3C1	ASAP2	OAZ1	DDX41		

	YES1	HSP90AB1	MRPL9	NCKAP5		
	CDC42	GFPT1	GABBR1	ZBTB25		
	HSP90B1	TF	RABGEF1	EFS		
	MED1	ATXN10	ZNF273	EXTL3		
	ICAM1	NFS1	GIT2	SHROOM2		
	SLC25A5	VAPA	DARS2	SETD2		
	KHDRBS1	TMCO3	EFEMP1	USO1		
	MED14	FCGR2B	CBR1	DUSP15		
	TGFA	MUC1	SOS1	HIST1H3E		
	CPS1	SFN	LRRK1	SQRDL		
	PKM2	C15ORF48	LGALS3	PCDHA7		
	MAP2K4	CTNND1	MED22	HIST1H3D		
	KRT17	PTPRO	ARRB2	CCKBR		

Supplementary Table 4. The relationship among STUB1, GPX4 expression and the clinicopathologic features.

Factors	STUB1			GPX4		
	Low	High	P-value	Low	High	P-value
Sex						
Female	92(41.4%)	87(44.4%)	0.544	68(39.1%)	111(45.5%)	0.192
Male	130(58.6%)	109(55.6%)		106(60.9%)	133(54.5%)	
Age(years)						
≤60	114(51.4%)	100(51.0%)	0.946	95(54.6%)	119(48.8%)	0.240
>60	108(48.6%)	96(49.0%)		79(45.4%)	125(51.2%)	
Location						
Gastric	170(76.6%)	101(51.5%)	*<0.001*	124(71.3%)	50(20.5%)	*0.020*
Non-Gastric	52(23.4%)	95(48.5%)		147(84.5%)	97(39.8%)	
Tumor size						
≤5cm	116(52.3%)	121(61.7%)	0.051	90(51.7%)	147(60.2%)	0.083
>5cm	106(47.7%)	75(38.3%)		84(48.3%)	97(39.8%)	
Mitotic index						
≤5/50HPF	130(58.6%)	149(76.0%)	*<0.001*	105(60.3%)	174(71.3%)	*0.019*
>5/50HPF	92(41.4%)	47(24.0%)		69(39.7%)	70(28.7%)	
NIH risk grade						
Very low-low	79(35.6%)	101(51.5%)	*0.001*	61(35.1%)	119(48.8%)	*0.005*
Moderate-high	143(64.4%)	95(48.5%)		113(64.9%)	125(51.2%)	
Morphology						
Spindle	193(86.9%)	182(92.9%)	*0.047*	156(89.7%)	219(89.8%)	0.974
Epithelioid and Mixed	29(13.1%)	14(7.1%)		18(10.3%)	25(10.2%)	
Mutation type						
Exon 11	203(91.4%)	172(87.8%)	0.216	152(87.4%)	223(91.4%)	0.180
Exon 9,13,17	19(8.6%)	24(12.2%)		22(12.6%)	21(8.6%)	
STUB1						
Low	-	-	-	128(73.6%)	94(38.5%)	*<0.001*
High				46(26.4%)	150(61.5%)	
GPX4						
Low	128(57.7%)	46(23.5%)	*<0.001*	-	-	-
High	94(42.3%)	150(76.5%)				

All of our variables with p-values less than 0.05 are in italics. HPF: high-power fields; NIH: National Institutes of Health.

Supplementary Table 5. Univariate and multivariate Cox regression analysis of STUB1, GPX4 expression and clinicopathological variables predicting the recurrence-free survival in patients with gastrointestinal stromal tumor.

Factors	Univariate		Multivariate	
	HR (95% CI)	P value	HR (95% CI)	P value
Sex				
Female	1	0.604	-	-
Male	1.201 (0.601-2.398)			
Age(years)				
≤60	1	0.071	-	-
>60	1.913 (0.947-3.866)			
Location				
Gastric	1	0.536	-	-
Non-Gastric	1.241 (0.627-2.456)			
Tumor size				
≤ 5cm	1	0.061	-	-
>5cm	1.919 (0.969-3.799)			
Mitotic index				
≤ 5/50HPF	1	*<0.001*	1	*<0.001*
>5/50HPF	4.382 (2.135-8.993)		3.907 (1.838-8.305)	
Morphology				
Spindle	1	*<0.001*	1	*0.046*
Epithelioid and Mixed	3.723 (1.780-7.788)		2.179 (1.013-4.685)	
Mutation type				
Exon 11	1	0.376	-	-
Exon 9,13,17	1.535 (0.594-3.965)			
STUB1				
Low	1	*0.039*	1	*0.011*
High	0.460 (0.220-0.962)		0.370 (0.172-0.794)	
GPX4				
Low	1	*0.006*	1	*<0.001*
High	3.420 (1.416-8.260)		5.806 (2.349-14.353)	

All of our variables with p-values less than 0.05 are in italics. HPF: high-power fields; NIH: National Institutes of Health.

Supplementary Table 6. Univariate and multivariate Cox regression analysis of STUB1, GPX4 expression and clinicopathological variables predicting the overall survival in patients with gastrointestinal stromal tumor.

Factors	Univariate		Multivariate	
	HR (95% CI)	P value	HR (95% CI)	P value
Sex				
Female	1	0.758	-	-
Male	0.735 (0.104-5.220)			
Age(years)				
≤60	1	0.305	-	-
>60	66.965 (0.022-207921.394)			
Location				
Gastric	1	0.652	-	-
Non-Gastric	0.594 (0.062-5.712)			
Tumor size				
≤ 5cm	1	0.786	-	-
＞5cm	1.312 (0.185-9.316)			
Mitotic index				
≤ 5/50HPF	1	0.122	-	-
＞5/50HPF	5.972 (0.621-57.411)			
Morphology				
Spindle	1	0.656	-	-
Epithelioid and Mixed	0.042 (0.000-47866.393)			
Mutation type				
Exon 11	1	0.662	-	-
Exon 9,13,17	0.042 (0.000-60861.505)			
STUB1				
Low	1	0.908	-	-
High	1.122 (0.158-7.965)			
GPX4				
Low	1	0.355	-	-
High	48.298 (0.013-178512.781)			

All of our variables with p values less than 0.05 are in italics. HPF: high-power fields; NIH: National Institutes of Health.
